# Myosin-actin crossbridge independent sarcomere length induced Ca^2+^ sensitivity changes in skinned myocardial fibers: Role of myosin heads

**DOI:** 10.1016/j.yjmcc.2025.03.003

**Published:** 2025-03-10

**Authors:** Xutu Wang, R. John Solaro, Wen-Ji Dong

**Affiliations:** a Voiland School of Chemical and Bioengineering, Washington State University, Pullman, WA 99163-1062, USA; b Department of Physiology and Biophysics, College of Medicine, University of Illinois at Chicago, Chicago, IL 60612-7342, USA; c Department of Integrative Physiology and Neuroscience, Washington State University, Pullman, WA 99163-1062, USA

**Keywords:** Length dependent activation, Cardiac muscle, Skinned myocardial fibers, Troponin I, Troponin C, Troponin T, Ca^2+^ sensitivity, Myosin-actin cross-bridge, Myosin-troponin interaction

## Abstract

Sarcomere length-dependent activation (LDA) is essential to engaging the Frank-Starling mechanism in the beat-to-beat regulation of cardiac output. Through LDA, the heart increases the Ca^2+^ sensitivity of myocardial contraction at a longer sarcomere length, leading to an enhanced maximal force at the same level of Ca^2+^. Despite its importance in both normal and pathological states, the molecular mechanism underlying LDA, especially the origin of the sarcomere length (SL) induced increase in myofilament Ca^2+^sensitivity, remains elusive. The aim of this study is to interrogate the role of changes in the state of myosin heads during diastole as well as effects of strong force-generating cross-bridges (XB) as determinants of SL-induced Ca^2+^ sensitivity of troponin in membrane-free (skinned) rat myocardial fibers. Skinned myocardial fibers were reconstituted with troponin complex containing a fluorophore-modified cardiac troponin C, cTnC(13C/51C)_AEDANS-DDPM_, and recombinant cardiac troponin I (cTnI) mutant, ΔSP-cTnI, in which the switch peptide (Sp) of cTnI was replaced by a non-functional peptide link to partially block the force-generating reaction of myosin with actin. We used the reconstituted myocardial fibers as a platform to investigate how Ca^2+^ sensitivity of troponin within skinned myocardial fibers responds to sarcomere stretch with variations in the status of myosin-actin XBs. Muscle mechanics and fluorescence measurements clearly showed similar SL-induced increases in troponin Ca^2+^ sensitivity in either the presence or the absence of strong XBs, suggesting that the SL-induced Ca^2+^ sensitivity change is independent of reactions of force generating XB with the thin filament. The presence of mavacamten, a selective myosin-motor inhibitor known to promote transition of myosin heads from the weakly actin-bound state (ON or disordered relaxed (DRX) state) to the ordered off state (OFF or super-relaxed (SRX) state), blunted the observed SL-induced increases in Ca^2+^ sensitivity of troponin regardless of the presence of XBs, suggesting that the presence of the myosin heads in the weakly actin bound state, is essential for Ca^2+^-troponin to sense the sarcomere stretch. Results from skinned myocardial fibers reconstituted with troponin containing engineered TEV digestible mutant cTnI and cTnT suggest that the observed SL effect on Ca^2+^ sensitivity may involve potential interactions of weakly bound myosin heads with troponin in the actin/Tm cluster region interacting with cTnT-T1 and residues 182–229 of cTnT-T2. The mechanical stretch effects may then be subsequently transmitted to the N-cTnC via the IT arm of troponin and the N-terminus of cTnI. Our findings strongly indicate that SL- induced potential myosin-troponin interaction in diastole, rather than strong myosin-actin XBs, may be an essential molecular mechanism underlying LDA of myofilament.

## 1. Introduction

Ca^2+^ regulation of cardiac function is complicated because multiple components of the sarcomere affect each other via multiple feedback mechanisms. Among them, sarcomere length-dependent activation (LDA) is essential for beat-to-beat regulation of cardiac output. Through LDA, the heart increases the responsiveness of myocardial contraction to activating [Ca^2+^] at a longer sarcomere length, leading to an enhanced tension generation at the same level of Ca^2+^. LDA has been considered the cellular basis underlying the Frank-Starling law of the heart. Loss of this dependence of contractions on sarcomere length (SL) has been identified as one of the hallmarks of failing hearts [1–5]. Despite its clinical significance, the mechanism underlying LDA remains poorly understood [6–9].

Implicit in findings that sarcomere stretch induces enhanced myofilaments Ca^2+^ sensitivity and maximal force development is the likely involvement of mechanisms activating the myofilament. Current theories indicate that both Ca^2+^ binding to cTnC and strong cross-bridge (XB) interactions with thin filament are essential components of activation and are modulated by sarcomere length. At the molecular level, Ca^2+^ binding to cTnC induces the cTnC N-domain (N-cTnC) to “open” [10,11] and to then strongly interact with the C-domain of cTnI (C-cTnI) [10–13]. The C-cTnI is the core component of the troponin regulatory switch and comprises the inhibitory region (Ir), switch peptide (Sp) and mobile domain (Md) [14–17]. The Ca^2+^-induced interaction between N- cTnC and the Sp of cTnI facilitates the shift of tropomyosin (Tm) from the blocked state to the closed state to promote strong binding of myosin motors to the actin filament [18–23]. Subsequent strong XB binding ultimately shifts Tm to the open state, induces further changes in troponin structure and enhances its Ca^2+^ sensitivity in what is termed “XB feedback” [20,24–29]. These Ca^2+^-/XB-induced structural transitions in troponin and Tm are the molecular basis of myofilament regulation and have been implicated in LDA via the cooperative activation mechanism [9,20]. Current wisdom suggests that enhancement of myofilament force upon sarcomere stretch is due to an increase in the number of force producing XBs in the cardiac sarcomere through a thick filament mediated mechanism [30–33], whereas the stretch induced increase in myofilament Ca^2+^ sensitivity is related to structural changes in the thin filament [34–36] caused by a feedback pathway through strongly bound myosin-actin XBs [37–40]. In addition, there is the theory that stretches of titin may affect XB state. Titin, which connects the Z disk to the M band and interacts with both the thick and the thin filaments of the cardiac sarcomere, is considered a length sensor for LDA via its compliance (passive tension development). At constant cell volume, titin stretch may lead to a decrease in interfilament lattice spacing in diastole promoting entry of cross-bridges into force production [41,42] by allowing myosin heads in weakly bound state to reach out to troponin for interaction. Real-time X-ray diffraction studies of both stretch-activated Lethocerus indirect flight muscles (IFM) [43] and twitching rat cardiac muscle [36] provide evidence of the communications between myosin and troponin.

New information has reshaped our understanding of processes at the level of thin, thick, and titin filaments and raised questions on how they contribute to the Frank Starling relation. Although it remains possible that the compression of the inter-filament spacing with increases in SL may promote interactions of cross-bridges with the thin filament, there is controversy regarding this hypothesis. The elements of this controversy have undergone thorough review with a summary of multiple findings listed by de Tombe et al. that are pro and con [7]. The main conclusion from the de Tombe et al. analysis and various experimental approaches is that rather than the spacing between the filaments being a unifying mechanism, LDA is related to mechanisms associated with an effect of SL on modulation of thick filament state, most likely by stretch of titin that signals thin filament activation thereby promoting the number force generating cross-bridges. Supporting evidence has been reported indicating that isoform switching of cTnI with slow skeletal TnI as well as post-translational modifications of sarcomere proteins such as MyBP-C3 and cTnI modulate LDA independent of changes in inter- filament spacing [7,44].

In the present manuscript we have designated XBs related to an interacting head motif (IHM) in an SRX (super relaxed state) or a DRX (disordered relaxed state) as is abundantly evident in the literature [45,46]. However, we are fully aware of the data demonstrating that this concept needs to be considered in the context of finding that do not fit with the idea of the interacting head motif (IHM) or the designation of SRX and DRX descript XB states [47,48]. In any case, related findings from multiple studies support the hypothesis that the mechanism underling SL-induced increase in myofilament Ca^2+^ sensitivity is associated with the number of myosin heads in the weakly bound state (DRX or ON), rather than the number of strong myosin-actin cross- bridges. These studies reported that the number of XBs in the DRX state in isolated skinned myocardium increases as sarcomere is stretched [37,38,49–51]. This hypothesis is also supported by a recent study, in which the SL-induced changes in troponin were observed in the presence of blebbistatin to inhibit entry of myosin motors into strong XB binding in skinned myocardial fibers [35].

Interestingly, this idea of strong XBs activating the thin filament has been turned around with evidence that in early Ca^2+^ activation of sarcomeres, so called “sentinel” cross-bridges in the thick filament produce enough force to induce a strain that is transmitted along the thick filament promoting the motors entering the force generating state most likely by an initially transferring to an intermediate weakly bound DRX state [52–54]. A critical element in this strain dependent activation is that there is a variable “gain” in this strain. In direct determinations of mechano-sensitivity of thick filament strain by atomic force microscopy, stretching myofibrillar titin has been demonstrated to increase lateral strain on the A-band independent of changes in lattice spacing [55]. There is also recent evidence of variation in thick filament strain as detected by structural signals obtained by nano-meter x-ray diffraction studies comparing fast skeletal muscle to cardiac muscles [56]. Although the evidence suggests that sensing of stretch via titin to troponin/thin filament Ca^2+^ activation mechanisms via altered thick filament state, detailed signaling pathways and potential protein–protein interaction underlying the mechanism are unknown.

In this study we aim to investigate the molecular basis of this SL- induced potential myosin-troponin interaction to test a hypothesis that SL induced mechano-signaling allows direct interaction between the troponin and the myosin heads released from super relaxed state (SRX) by SL stretch [30,53], which allosterically sensitizes troponin for Ca^2+^ binding at diastolic levels. However, there is a significant technical challenge to test the hypothesis. For myocardial contraction the Ca^2+^- activated myosin-actin XB interaction is a dominant event during force development and can allosterically affect thin filament regulation pathways [37–40,57,58]. It is known that multiple myosin heads can interact with each regulatory unit of the thin filament comprising 1 troponin, 1 Tm and 7 actin proteins. Therefore, a key challenge for experimentally examining the SL-induced potential troponin-myosin interaction in myocardial study is to distinguish the effect of the potential 1:1 myosin-troponin interaction from the effects generated by multiple dominant myosin-actin XB interactions.

To address this challenge, we designed a unique recombinant cTnI mutant ΔSP-cTnI, in which the Sp (residues 152–167) of cTnI are replaced by a non-functional link sequence (GGGS)_3_ [59], to block actin- myosin XB interactions of skinned myocardial fibers. Without functional Sp, the ΔSP-cTnI will strongly bind to the actin filament and constrain Tm in the blocked-state, inhibiting myosin-actin interactions of myocardium regardless of the Ca^2+^ activation level of the myofilament. Using skinned myocardial fibers from rat heart reconstituted with troponin complex containing fluorophore-modified cTnC(13C/51C)_AEDANS-DDPM_ and ΔSP-cTnI as a platform, we can investigate how calcium sensitivity of troponin within skinned myocardial fibers responds to sarcomere stretch without interferences from myosin-actin XB interactions. Ca^2+^ sensitivity of troponin was detected by monitoring N- domain opening of cTnC(13C/51C)_AEDANS-DDPM_ within skinned myocardial fibers in response to Ca^2+^ titration using in situ fluorescence resonance energy transfer (FRET) technique developed in our lab [34,60]. Since opening of the N-cTnC requires both Ca^2+^binding and the presence of Sp of cTnI [10,11], a synthesized Sp peptide of cTnI was used in the Ca^2+^-titration experiments to monitor Ca^2+^ sensitivity changes of opening of the N-cTnC in response to SL stretch. Our results clearly demonstrate that the SL-induced Ca^2+^ sensitivity change is independent of strong myosin-actin XB but requires myosin heads in weakly bound (DRX) state. The SL effect on Ca^2+^ sensitivity may involve the potential interaction of the myosin heads with troponin in the region composing of residues 180–276 of cTnT. However, we cannot rule out alternative mechanisms.

## 2. Results

### 2.1. Western blot analysis of the reconstituted skinned cardiac muscle fibers

**Fig. 1.**
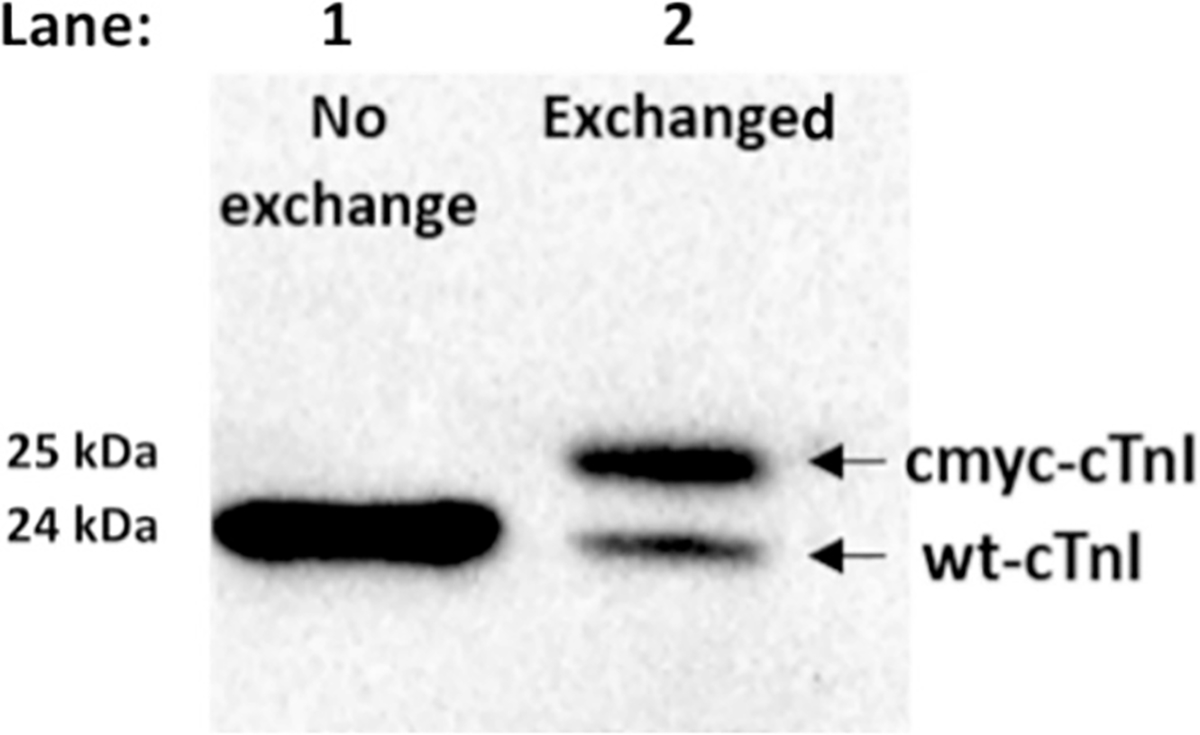
Western blot analysis of skinned myocardial fibers before (lane 1) and after (lane 2) reconstitution with the troponin complex containing wt-cTnT, cMyc-cTnI, and cTnC(13C/51C)_AEDANS-DDPM_. Lane 1 shows a single band at ~24 kDa of the intrinsic wt-cTnI protein extracted from the non-exchanged skinned fibers. Lane 2 shows two bands, one at ~24 kDa of the wt-cTnI and the other at ~25 kDa of the cMyc-cTnI protein extracted from the protein- exchanged skin fibers. The cMyc-tag is around 1 kDa. The protein exchange efficiency of this protocol reached 80 ± 5 % and it was analyzed by ImageLab density analysis software. Mouse monoclonal anti-cTnI antibody (Cat: ab10231, Abcam) and HRP-labeled sheep anti-mouse secondary antibody (GE Healthcare) were used in this experiment.

To assess our troponin complex-based reconstitution protocol for skinned cardiac muscle fibers, we selected cTnI as a marker protein to examine the efficiency of troponin protein exchange using Western blot assay. We used troponin complex containing cMyc-cTnI (~ 25 kDa) to replace the intrinsic wt-cTnI (~ 24 kDa) in the muscle fibers. The cMyc was tagged to the N-terminus of cTnI. Comparing tension measurements vs. Ca^2+^ titration of skinned myocardial fibers reconstituted with cMyc- tagged cTnI and wild-type cTnI (data not shown) indicated no significant functional effect of the tag on muscle fiber contractions. Because of the differences in their molecular weights, cMyc-cTnI tagged with cMyc sequence of EQKLISEEDL (1.2 kDa) and the intrinsic wt-cTnI can be easily distinguished on Western blot gel for estimate of the exchange ratio. Fig. 1 shows the result of Western blot analysis of the non- reconstituted and reconstituted skinned muscle fibers with troponin complex containing cMyc-cTnI. For the non-exchanged skinned muscle fibers, a single protein band at 24 kDa, indicating the presence of the endogenous wt-cTnI protein, was observed. For the protein-exchanged fibers, two protein bands were observed at 24 kDa and 25 kDa, respectively, indicating the presence of both the endogenous wt-cTnI and cMyc-cTnI proteins. An 80 ± 5 % exchange ratio of cMyc-cTnI to endogenous wt-cTnI of the reconstituted fibers was estimated by quantifying densities of the two bands using ImageLab software (Bio-Rad). The observed exchange efficiency is consistent with our previous studies using the same protocol [61].

### 2.2. Effects of SL on Ca^2+^ sensitivity of skinned myocardial fibers in the presence/absence of strong XBs

**Fig. 2.**
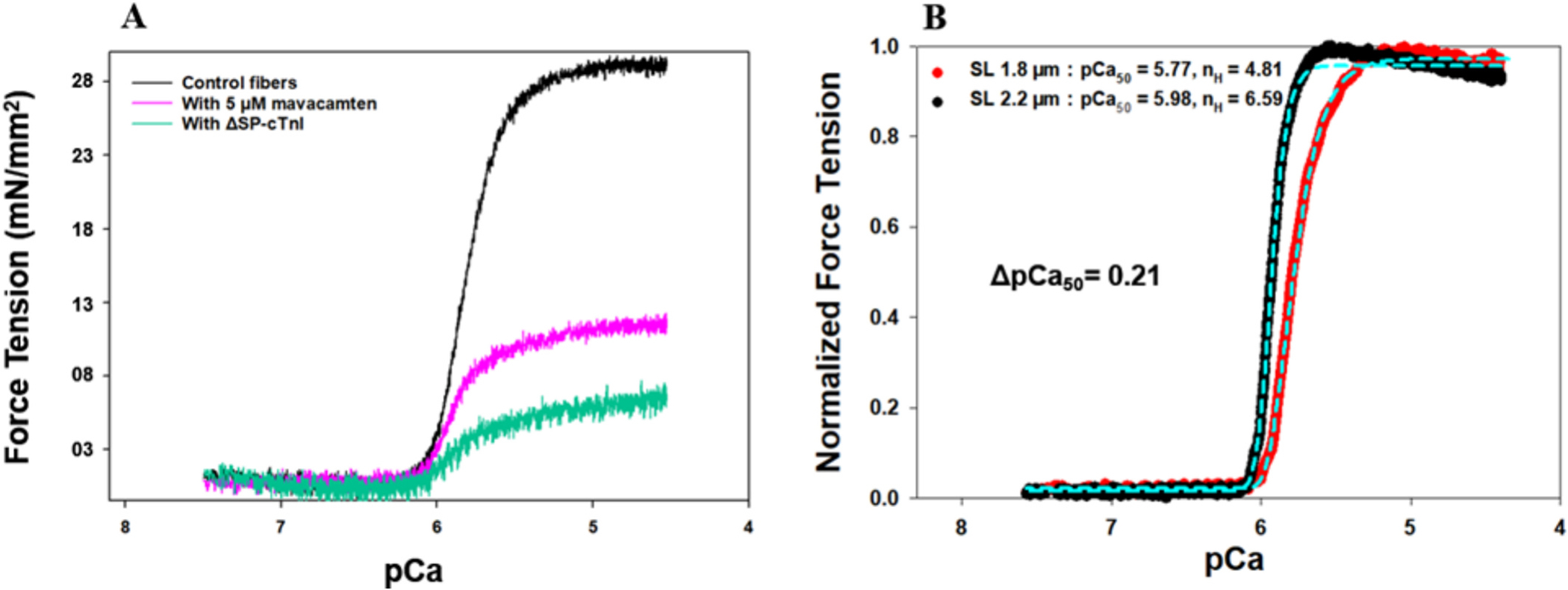
Tension development vs. Ca^2+^ titration of skinned myocardial fibers under different conditions. A: Activated tensions of the skinned myocardial fiber reconstituted with troponin containing ΔSP-cTnI reconstitution (teal) and wt-cTnI in the absence (black) and presence (pink) of 5 μM of mavacamten. B: Normalized tension development vs. Ca^2+^ titration at SL 1.8 (red) and 2.2 μm (black) of skinned myocardial fibers reconstituted with Group I troponin complex containing cTnC (13C/51C)_AEDANS-DDPM_, wt-cTnT, and cMyc-cTnI. Data (solid lines) were fit to a 4-parameter Hill equation (cyan dash lines) to derive the Ca^2+^ sensitivity (pCa_50_) and slope n_H_ (the Hill coefficient) at different SLs, which are given in the legend. (N_fiber_ = 6 for B, the curves shown in both A and B represent the average curves of all measurements, and the errors calculated from STD are shown in Fig. 5 and Table 1). The maximal Ca^2+^ activated forces corresponding to tension-Ca2+ titrations shown in panel B were 28.9 mN/mm^2^ for SL of 2.2 μm and 21.3 mN/mm^2^ for SL of 2.8 μm, respectively.

The objective of this experiment was to examine how SL affects the Ca^2+^ sensitivity of skinned myocardial fibers in the absence of myosin- actin XBs. To eliminate XB formation between actin and myosin heads, we replaced the intrinsic wt-cTnI of skinned myocardial fibers with the mutant ΔSP-cTnI using our reconstitution protocol [62]. Substitution of the switch peptide (Sp) sequence (152–167) with a nonfunctional linker (GGGS)_3_ enables the ΔSP-cTnI to stay at its inhibition position on the actin filament regardless of the Ca^2+^binding, thus, inhibiting the Ca^2+^-activated force generating XBs in the reconstituted myocardial fibers. The inhibition efficiency of the ΔSP-cTnI on strong XBs was investigated by measuring the Ca^2+^-induced force changes of the ΔSP-cTnI reconstituted myocardial fibers. The force contraction of the fibers reconstituted with ΔSP-cTnI was reduced by approximately 80 ± 2.8 % compared to the fibers reconstituted with wt-cTnI (Fig. 2A), suggesting that lack of the Sp sequence in cTnI abolished Ca^2+^-activated contractility of the skinned muscle fibers by inhibiting actin-myosin binding. Previously, several studies used recombinant cTnC mutant, in which the Ca^2+^-binding site II was inactivated, to prevent strong actin- myosin interactions; exchanged chemically skinned muscle fibers with these cTnC mutants reduced activation force by 80–90 % [63–66]. This result is consistent with what we observed in the present study.

To examine SL-induced changes in Ca^2+^ sensitivity of the thin filament of myocardial fibers in response to actin-myosin XB interactions, we prepared two groups of reconstituted skinned rat myocardial fibers. *Group I* fibers were reconstituted with a troponin complex containing cTnC(13C/51C)_AEDANS-DDPM_, wt-cTnT, and cMyc-cTnI to allow normal actin-myosin XB interactions, and *group II* fibers were reconstituted with a troponin complex containing cTnC(13C/51C)_AEDANS-DDPM_, wt-cTnT, and ΔSP-cTnI to inhibit myosin-actin XB interactions. The Ca^2+^- dependent structural opening of the cTnC N-domain, which is proportional to the Ca^2+^ sensitivity of troponin/thin filament of myocardial fibers, was determined by monitoring the change in FRET fluorescence intensity between the donor (AEDANS) and the acceptor (DDPM) on cTnC(13C/51C)_AEDANS-DDPM_ in response to Ca^2+^ titration at different SL [61].

**Fig. 3.**
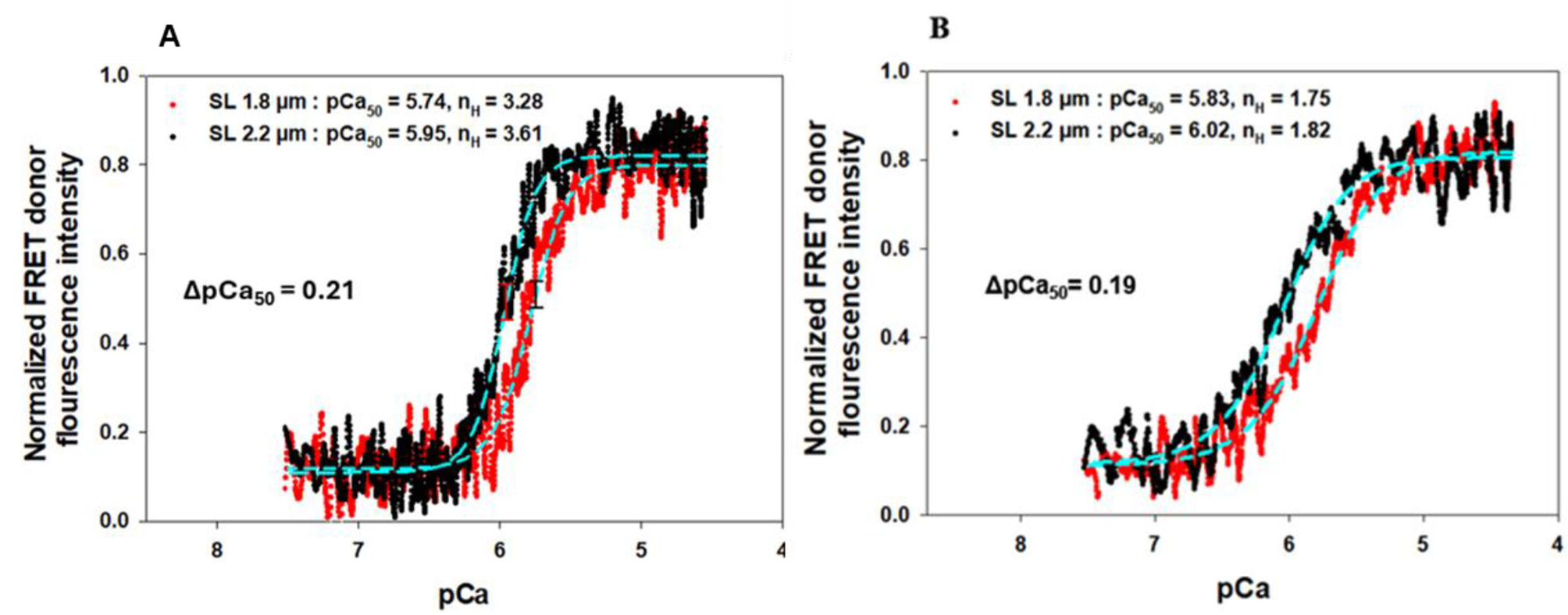
The normalized FRET donor fluorescence intensity changes vs. Ca^2+^ titration at SL 1.8 (red) and 2.2 μm (black) in skinned myocardial fibers reconstituted with Group I complex containing cTnC(13C/51C)_AEDANS-DDPM_, wt-cTnT, and cMyc-cTnI (A) and Group II containing cTnC(13C/51C)_AEDANS-DDPM_, wt-cTnT, and ΔSP- cTnI (B). Data (dots) were fit to a 4 parameter Hill equation (cyan dash lines) to derive the Ca^2+^ sensitivity (pCa_50_) and n_H_ (the Hill coefficient) at different SLs, which are listed in the legend. (N_fiber_ = 6 for A, and N_fiber_ = 6 for B, the curves shown in both A and B represent the average curves of all measurements, and the errors calculated from STD are shown in Fig. 5 and Table 1.)

Fig. 3 shows the FRET donor fluorescence intensity changes of both the reconstituted rat myocardial fiber *group I (A)* and *II (B)* vs. Ca^2+^ titrations from pCa 8.3 to pCa 4.3 at SL of 1.8 μm and 2.2 μm, respectively. The binding of Ca^2+^ to the N-domain of cTnC causes a conformational change, opening the domain and exposing a hydrophobic site for the cTnI-SP to bind [10,67]. The Ca^2+^-induced conformational opening of the N-domain of cTnC separates the donor AEDANS from the acceptor DDPM, thus increasing the fluorescence intensity of the donor AEDANS. For the reconstituted myocardial fiber *group I* sample, normalized force developments of the fibers vs. Ca^2+^ titrations from pCa 8.3 to pCa 4.3 at both short and long SLs were also measured and shown in Fig. 2B. Each of the force and FRET titration curves was fitted with the Hill equation to derive the *pCa*_*50*_ and the Hill coefficients (*n*) as described previously [61,68]. The obtained results are summarized in Table 1.

For the *group I* fibers with the wt-cTnI, which allows the active myosin-actin XB formations, the *pCa*_*50*_ derived from FRET-Ca^2+^ titrations (Fig. 3A.) were 5.74 ± 0.03 and 5.95 ± 0.04 at 1.8 and 2.2 μm SL, and respectively, amounting to a SL-induced change of 0.21 in *pCa*_*50*_. This SL-induced change of *ΔpCa*_*50*_ was consistent with the *ΔpCa*_*50*_ obtained from the Ca^2+^-force titrations shown in Fig. 2B. The Hill coefficient (*n*) associated with the Ca^2+^-induced conformational change of the troponin complex increased from 3.28 to 3.61 upon SL increase (Table 1), suggesting a more cooperative binding of Ca^2+^ at longer SL. The observed cooperativities of Ca^2+^-induced troponin conformational change are lower than the cooperativities of Ca^2+^-activated force development. Most likely, this difference is due to the multiple levels of protein-protein interactions involved in myocardial force development.

When the ΔSP-cTnI was used in the *Group II* reconstitution fibers, the maximum Ca^2+^-activated force was significantly reduced by more than 80 ± 2.8 % (Fig. 2A). However, the N-domain opening of the cTnC could still be activated by the extrinsic Sp peptide and the Ca^2+^ ion, and the Ca^2+^-induced fluorescence intensity changes are shown in Fig. 3B. The derived *pCa*_*50*_ of N-cTnC opening was 5.83 ± 0.05 and 6.02 ± 0.06 at 1.8 and 2.2 μm SL, respectively, amounting to a change of 0.19 in Δ*pCa*_*50*_. When comparing the pCa_50_ values at short and long SLs of *group I* fibers to *group II* fibers, respectively, they both shifted to the right from 5.74 ± 0.03 to 5.83 ± 0.05 at 1.8 μm SL and from 5.95 ± 0.04 to 6.02 ± 0.06 at 2.2 μm SL, respectively. These shifts could be caused by the presence of extrinsic SP in *group II* fibers to synergize the Ca^2+^-induced opening of N-cTnC. In addition, compared to the results from *group I*, the cooperativity of Ca^2+^-induced myofilament activation in *group II* sample was decreased, as suggested by the changes in the Hill coefficient (*n*) from 3.28 to 1.75 at short SL and from 3.61 to 1.82 at long SL (Table 1). The observed decreases in cooperativity likely resulted from the disengagement between thin filament and thick filament caused by ΔSP-cTnI, which is corroborated by a study of skeletal muscle force development using different TnC constructs for partial inactivation of thin filaments [58].

The surprising finding from this experiment was that there was no significant difference in the SL-induced ΔpCa_50_ derived from the *group I* and *group II* fibers. This observation is not consistent with the current theories that the myosin-actin XB plays a primary role in the SL-induced changes of Ca^2+^ sensitivity on the thin filament [7]. This result suggests that an alternative pathway, e.g., direct troponin-myosin interaction or mechano-sensitive mechanisms in the thick filament, may exist to transduce the mechanical stretch of titin in the sarcomere to the Ca^2+^ sensitivity changes of the thin filament. In the next experiment, we investigated whether the activation status of the myosin heads is involved in an alternative pathway, possibly involving supporting the troponin-myosin bridge hypothesis.

### 2.3. Effects of states of myosin heads on SL-induced changes in Ca^2+^ sensitivity of skinned myocardial fibers in the presence/absence of strong XBs

**Fig. 4.**
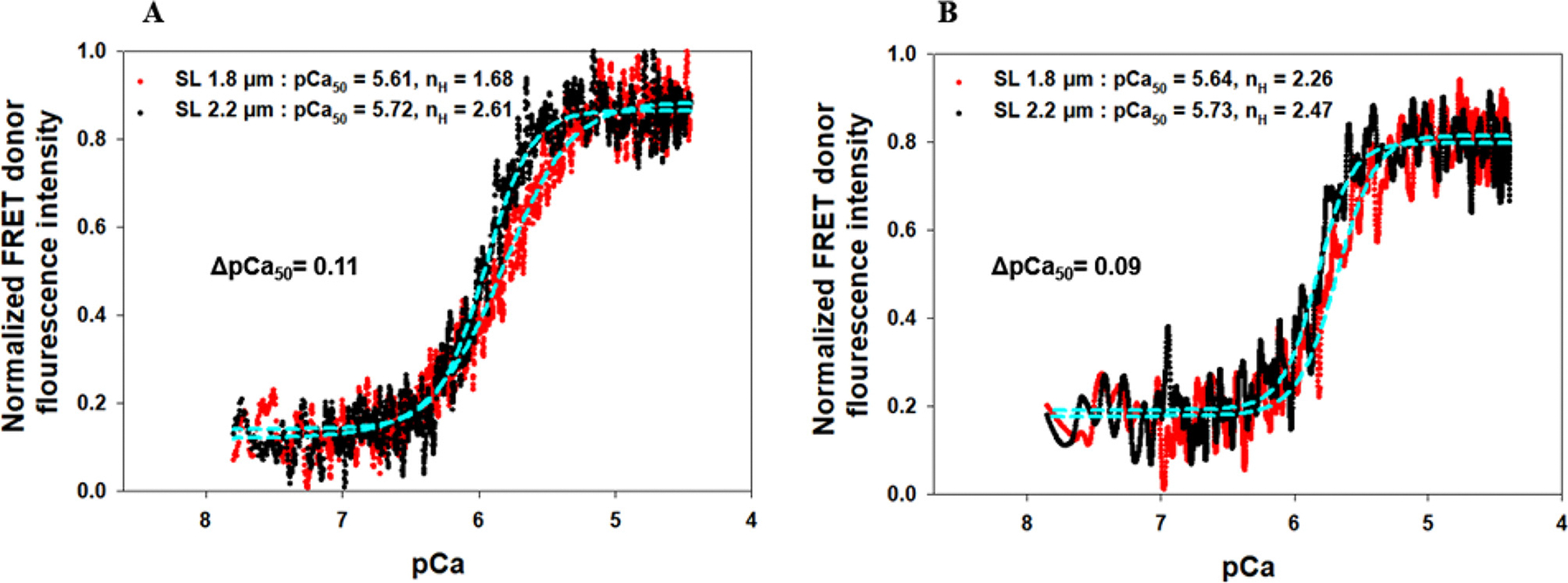
The normalized FRET donor fluorescence intensity changes vs. the Ca^2+^ titration at SL 1.8 (red) and 2.2 μm (black) of the myocardial fibers reconstituted with Group I complex containing cTnC(13C/51C)_AEDANS-DDPM_, wt-cTnT, and cMyc-cTnI (A) and Group II containing cTnC(13C/51C)_AEDANS-DDPM_, wt-cTnT, and ΔSP-cTnI (B) in the presence of 5 μM mavacamten drug. Data (dots) were fit to a 4 parameter Hill equation (cyan dash line) to derive the Ca^2+^ sensitivity (pCa_50_) and slope n_H_ (Hill coefficient) at different SLs, which are listed in the legend. (N_fiber_ = 5 for A, and N_fiber_ = 6 for B, the curves shown in both A and B represent the average value of all measurements, and the errors calculated from STD are shown in Fig. 5 and Table 1.)

It is known that myosin can exist in a low-energy consuming “super- relaxed” (SRX) state, and a the weakly bound “disordered relaxed” (DRX) state [52,69]. Sarcomere stretch induced myosin state transitions from SRX state to DRX state is critical for myosin heads to reach actin, which ultimately leads to an increased thin filament Ca^2+^ sensitivity and maximum force generation of myocardium [52,70]. In this experiment, we examined how transitions in myosin state affected the SL-induced Ca^2+^ sensitivity change in the presence/absence of the strong XBs of skinned myocardial fibers. Because of its primary role in increasing and stabilizing the population of SRX of cardiac myosin [71], 5 μM mavacamten was applied to both group fibers in this experiment to manipulate the populations of the DRX/SRX states of the myosin heads of the skinned myocardial fibers. The maximum Ca^2+^-activation force observed was reduced by 62 ± 4.1 % (Fig. 2A), which is consistent with the role of mavacamten to constrain the myosin heads at a low energy configuration, thus, reducing the myocardial contractility [71]. To examine how mavacamten affects the SL-induced Ca^2+^ sensitivity changes in skinned myocardial fibers, 5 μM mavacamten drug was applied to *group I* skinned myocardial fibers reconstituted with wt-cTnT, cMyc-cTnI, and cTnC(13C/51C)_AEDANS-DDPM_, and *group II* skinned fibers reconstituted with wt-cTnT, ΔSP-cTnI, and cTnC(13C/51C)_AEDANS-DDPM_ each. FRET donor fluorescence intensity changes vs. Ca^2+^ titration were performed on both groups at different sarcomere lengths shown in Fig. 4.

Each titration curve was fitted with the Hill equation, and the derived *pCa*_*50*_ value and Hill coefficients (*n*) were summarized in Table 1. With the group I fibers, the XB interactions were active, and the pCa_50_ was 5.61 ± 0.06 at 1.8 μm SL and 5.72 ± 0.03 at 2.2 μm SL. Comparing the pCa_50_ obtained from the same reconstituted fiber group with and without the drug (Table 1), the presence of 5 μM drug caused the myofilament activation to be less sensitive to Ca^2+^, i.e., reducing the pCa_50_ by 0.13 units at short SL and 0.23 unit at long SL. The use of the mavacamten drug also reduced the SL-induced ΔpCa_50_ of the fibers by 52.3 % (Table 1), from ΔpCa_50_ of 0.21 (without the drug) to 0.11 (with the drug). These significant drug-induced shifts in Ca^2+^ sensitivity are consistent with previous observations in force measurements of the skinned muscle fibers [69]. The observed drug effects are likely associated with the function of the drug, i.e., to constrain myosin heads in SRX state and reduce DRX population leading to less myosin-actin XB interactions. The observed drug-induced reduction in troponin cooperativity (*n*) for both sarcomere lengths (Table 1), as well as the SL-induced pCa_50_ shifts (ΔpCa_50_), are likely a consequence of this drug-induced decrease in XB interactions.

With the *group II* fibers (reconstituted with ΔSP-cTnI), in which the strong XB interactions were inhibited, the presence of mavacamten induced a similar trend of changes in the Ca^2+^ sensitivity of the fibers. pCa_50_, measured by the FRET-Ca^2+^ titration, was 5.64 ± 0.05 at 1.8 μm SL and 5.73 ± 0.04 at 2.2 μm SL (Fig. 4B). Comparisons of the pCa_50_ results obtained from the same group of fibers with and without the drug (Table 1), demonstrated that the presence of 5 μM drug caused the myofilament activation to be less sensitive to Ca^2+^, i.e., reducing pCa_50_ by 0.1 units at short SL and 0.29 units at long SL. This was accompanied by an increase in cooperativity of the troponin conformational change from an *n* of 1.75 to 2.26 at the short SL and from an *n* of 1.82 to 2.47 at the long SL. The presence of mavacamten reduced the SL activation on the pCa_50_ of ΔSP-cTnI reconstituted fibers by more than 47.4 % (Table 1), from ΔpCa_50_ of 0.19 (without drug) to 0.1 (with the drug). Same to the results that were observed in the g*roup I* fibers (reconstituted with cMyc-cTnI), these observed changes are likely caused by the drug- induced shift in the myosin population from DRX state to the SRX state, suggesting that the population of myosin heads in DRX state can modulate the impact the Ca^2+^ sensitivity of thin filament, as well as the SL effect on the Ca^2+^ sensitivity.

**Fig. 5.**
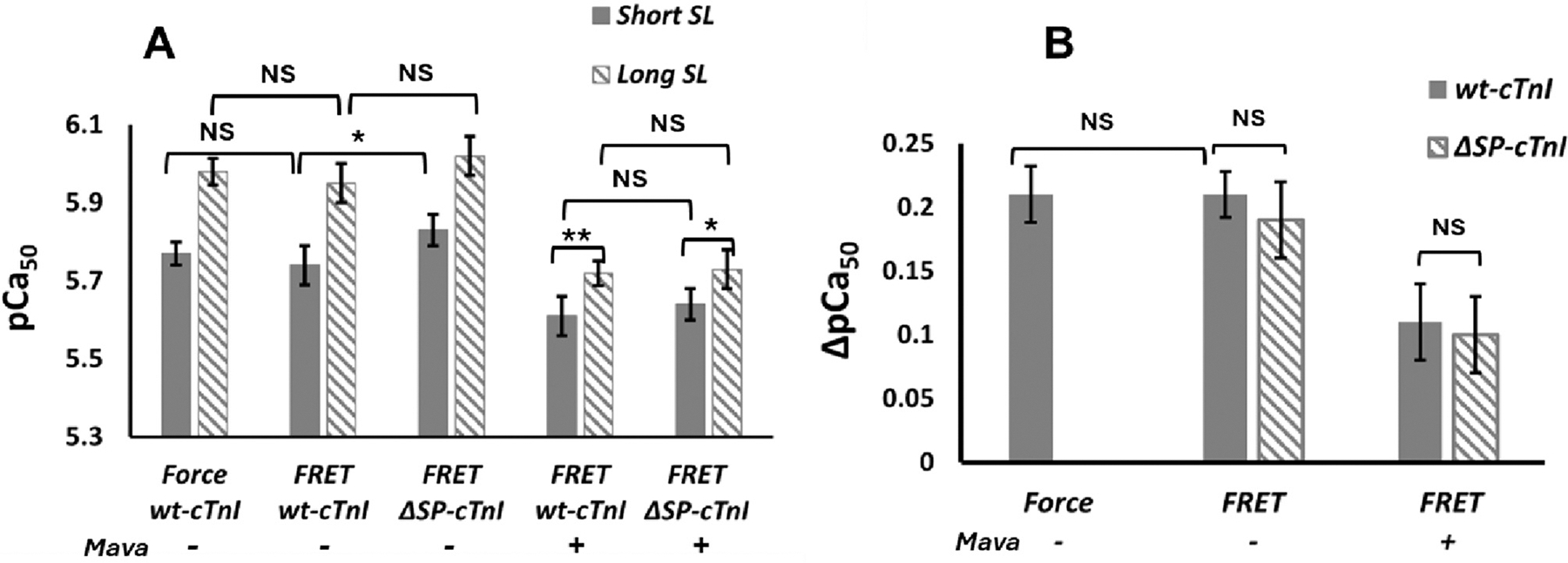
Collective results of pCa_50_ (A) and ΔpCa_50_ (B) measured by force- and FRET-Ca^2+^ titrations under different cTnI reconstitution conditions in the absence (−) and presence (+) of mavacamten at short SL (filled bar) and long SL (diagonal stripes bar). Values are reported as mean ± SD. **, *p* < 0.01, *, *P* < 0.05, NS, no significant changes.

Fig. 5 shows the collective results of pCa_50_ and ΔpCa_50_ obtained by force- and FRET-Ca^2+^ titrations under different troponin reconstitution conditions and different SLs in the absence (−) and presence (+) of mavacamten. Since the strong XB interactions as well as the XB feedback mechanism are not present in the group II fibers, the mechanism underlying the observed changes in the presence of mavacamten drug is unlikely to be associated with strong XB interactions; instead, these results supported our hypothesis that the direct troponin-myosin interaction may be involved, and the myosin heads at RDX state are likely required for this interaction. However, this experiment cannot fully define the nature of the proposed troponin-myosin interaction. In the next experiment, we aimed to obtain more information on how the troponin-myosin interaction or other interfilament signaling transduces mechanical signals to regulate the troponin Ca^2+^ sensitivity.

### 2.4. Effects of integrity of troponin structure on SL-induced changes in Ca^2+^ sensitivity of skinned myocardial fibers in the presence/absence of myosin-actin XBs

This experiment was designed to obtain more structural information on the proposed myosin and troponin interaction, specifically on which region of the troponin complex is likely involved in the interaction to transduce the sarcomere stretching to the Ca^2+^ sensitivity of troponin within skinned myocardial fibers. To achieve this goal, TEV protease digestible recombinant mutant TEV-cTnT, TEV-cMyc-cTnI, and TEV- ΔSP-cTnI were designed and used in this experiment.

**Graph 1.**
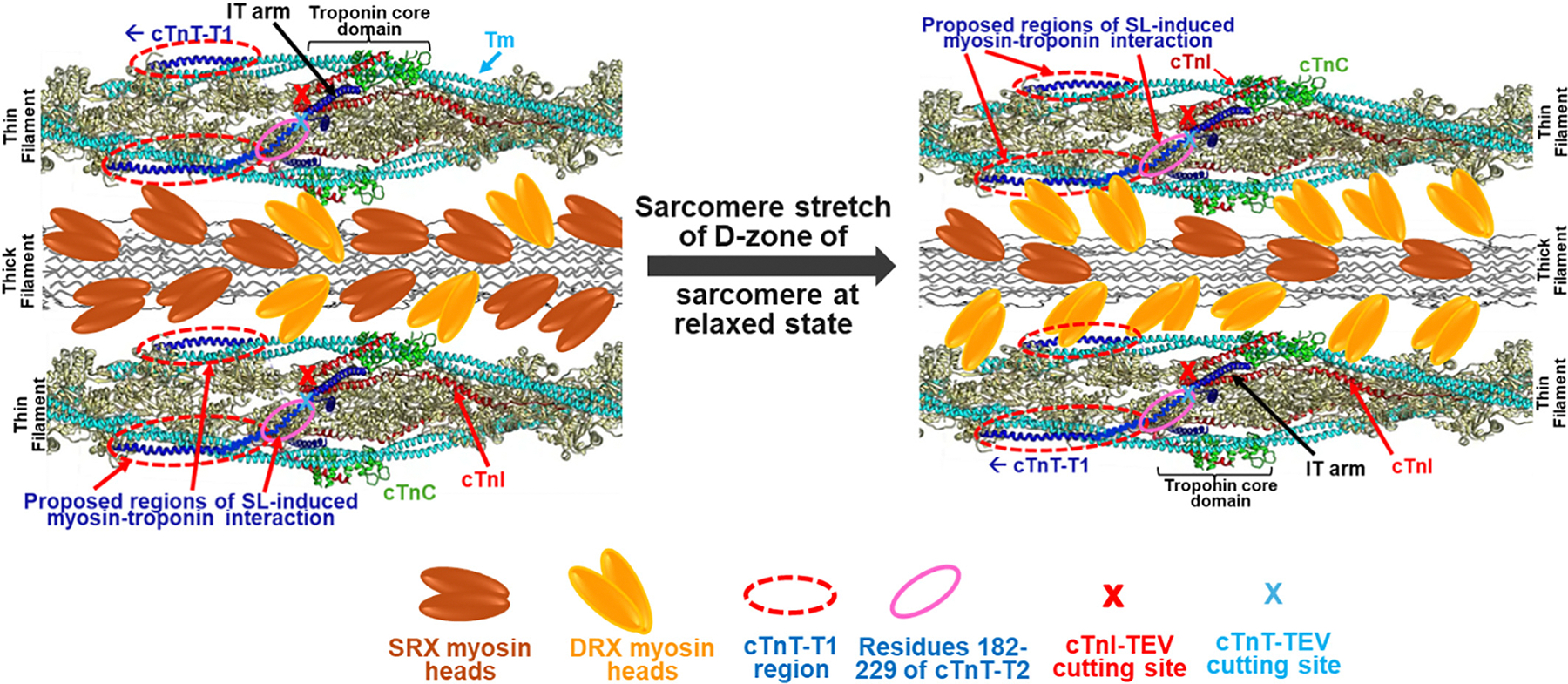
The schematic picture of the troponin bridge mechanism underlying sarcomere length dependent activation of the D-zone of sarcomere. The picture on the left shows the relaxation sarcomere at short SL, and the one on the right shows the relaxation sarcomere at long SL which interfilament space decreased. Myosin heads in SRX and DRX, TEV cutting site for cTnI and cTnT, and potential regions for SL-induced interaction between DRX myosin heads and troponin are all shown as indicated. The shown thin filament structure composed of cTnC, cTnI, cTnT, tropomyosin and actin filament are from literature (ref??) with minor modification to show the potential region of myosin heads and troponin interactions.

In the TEV-cTnT design, the TEV enzyme cutting sequence ENLYFQA was introduced to replace the sequence of residues 214–220 (sequence ERRKVLA) of wt-cTnT. cTnT comprises the T1 domain (residues 1–181 of the N-terminal region) and the T2 domain (residues 182–288 of the C- terminal region). The two domains are functionally and structurally different [72,73]. T1 anchors the troponin complex to the thin filament by strongly binding to Tm through its Tm binding site 1. The C-terminal region (residues 226–276) of T2 forms a structure termed the IT arm with cTnI (residues 90–136), which further interacts with the C-domain of cTnC [74]. The C-terminal region (residues 189–225) provides a second Tm binding site for cTnT-Tm-actin interactions and is considered the critical hinge region to link the troponin core domain to Tm/actin as illustrated in Graph 1. The inserted TEV sequence in the region of residues 214–220 was located within this region. Because this insertion region is located before the IT arm structure [74], no significant effect on IT structure by the insertion is expected. However, breaking this region will separate the core domain of the troponin complex from the T1/tropomyosin binding, which may affect transduction of stretch mechanic signals.

In the TEV-cTnI design, the TEV enzyme cutting sequence of ENLYFQA was introduced to replace the sequence of residues 84–90 (sequence LVLDGLG) of cTnI. This flexible region provides a link between the IT arm of the core domain of the troponin complex and the N- terminal region (residues 1–80) of cTnI that further extends its interaction with the N-domain of cTnC [74].

Our rationale for this experiment is that if the proposed troponin- myosin interaction is located at the core domain of the troponin or the IT arm region, TEV-cTnT cutting will have minimal effect on the SL- induced Ca^2+^ sensitivity changes in the presence of ΔSP-cTnI. Otherwise, the cTnT/Tm binding region may be involved in the interaction. Then, the effect of TEV-cTnI cutting will provide information on whether the signal is transduced to the N-domain of cTnC via the IT arm or the N-terminal region of cTnI.

Before performing Ca^2+^ sensitivity experiments on the reconstituted fibers, we examined the efficiency of in situ TEV digestions of TEV-cTnT and TEV-cTnI reconstituted myocardial fibers in heart relaxing (HR buffer). Western blot analysis shown in Fig. S1 indicates a 74 ± 3.2 % digestion rate for TEV-cTnT and 70 ± 4 % for TEV-cTnI after 4-h enzyme incubation of the fibers in the HR buffer. Western blot analysis was also performed in the HR buffer collected after enzyme digestion to detect the T2- cTnT fragment band at 27 kDa. However, no discernible band was observed from lane 5, suggesting that cTnT and cTnI are all retained within the sarcomere structure of the skinned fibers after TEV digestion.

To examine the nature of the proposed troponin-myosin interaction, we performed the force and FRET measurements vs Ca^2+^ titrations with the reconstituted skinned myocardial fibers under the following conditions.

- *Skinned rat myocardial fibers reconstituted with the troponin complex containing TEV-cTnT, cMyc-cTnI, and cTnC(13C/51C)*_*AEDANSE-DDPM*_ *with and without TEV enzyme incubations*. Figs. S2 and S3 show the normalized changes in force tension and FRET donor fluorescence intensity vs. the Ca^2+^ titration at short and long SL without and with TEV digestion, respectively. The pCa_50_ and the Hill cooperativity coefficient (*n*) derived for each condition are listed in Table 1. Without TEV cut, the pCa_50_ obtained from FRET-Ca^2+^ titration was 5.65 ± 0.05 at 1.8 μm SL and 5.83 ± 0.04 at 2.2 μm SL, which is a ΔpCa_50_ 0.20 (Fig. S2⋅B). Similarly, the SL-induced changes in tension development titration yielded 0.19 change of ΔpCa_50_ (Fig. S2A). These results are consistent with the results shown in Fig. 3A and Fig. 2B, respectively, suggesting TEV sequence insertion of cTnT does not alter the function of myocardial fibers. After TEV enzyme digestion in the presence of myosin-actin XB interactions, the SL- induced change in ΔpCa_50_ obtained from tension development vs. Ca^2+^ titration was reduced to 0.05 (Fig. S3A). The troponin conformational change, monitored by FRET fluorescence titration, also became significantly less sensitive to Ca^2+^ (Fig. S3B). The pCa_50_ at 1.8 μm SL and 2.2 μm SL was 5.73 ± 0.03 and 5.82 ± 0.03, respectively, which amounted to a 0.09 reduction in ΔpCa_50_. When the same FRET-Ca^2+^ titration experiments were performed with the skinned myocardial fibers reconstituted with the troponin complex containing TEV-cTnT, ΔSP-cTnI, and cTnC(13C/51C)_AEDANSE-DDPM_, in which the myosin-actin XB interaction was inhibited (Fig. S3C), TEV enzyme incubation also reduced the SL-induced ΔpCa_50_ to 0.08. These results showed that TEV enzyme cutting induced the same level of the reduction of the SL-induced ΔpCa_50_ changes regardless of the presence of myosin-actin XB interaction. The results strongly suggest that the hinge region of cTnT where the TEV sequence was inserted plays an important role in the signal transmission of sarcomere stretch to the N-cTnC and modulation of the Ca^2+^ sensitivity of troponin regulation. All changes of pCa_50_ and the Hill coefficient (n) derived from fitting the titration curves at different SLs are given in Table 1.
- *Skinned myocardial fibers reconstituted with the troponin complex containing wt-cTnT, cTnC(13C/51C)*_*AEDANSE-DDPM*_ *and TEV-cMyc-cTnI or TEV-ΔSP-cTnI with TEV enzyme incubations*. Fig. S4 shows the changes in normalized tension development (A) and FRET donor fluorescence intensity (B) vs. Ca^2+^ titration at different SLs when TEV-cMyc-cTnI, which allows active myosin-actin XB interaction, was used for skinned myocardial fiber reconstitution. Compared to the untreated samples, the TEV enzyme incubation led to a reduction of SL-induced ΔpCa_50_ changes from 0.19 to 0.20 to 0.05–0.06 (comparing Fig. S4A and Fig. S4B to Fig. 3), revealed by both force and FRET-Ca^2+^ titrations. When the XBs were inhibited in the reconstituted fibers containing TEV-ΔSP-cTnI, FRET-Ca^2+^ titration (Fig. S4C) showed that the pCa_50_ of the TEV treated reconstituted fibers shifted from 5.84 to 5.67 at SL 1.8 and shifted from 6.02 to 5.75 at SL 2.2, respectively, suggesting significant desensitization of Ca^2+^-N-cTnC binding at both short and long SL. SL induced ΔpCa_50_ also reduced from 0.19 to 0.08 (comparing Fig. 3A to Fig. S4C). The pCa_50_ and the Hill cooperativity coefficient (*n*) derived for each condition are listed in Table 1.

**Fig. 6.**
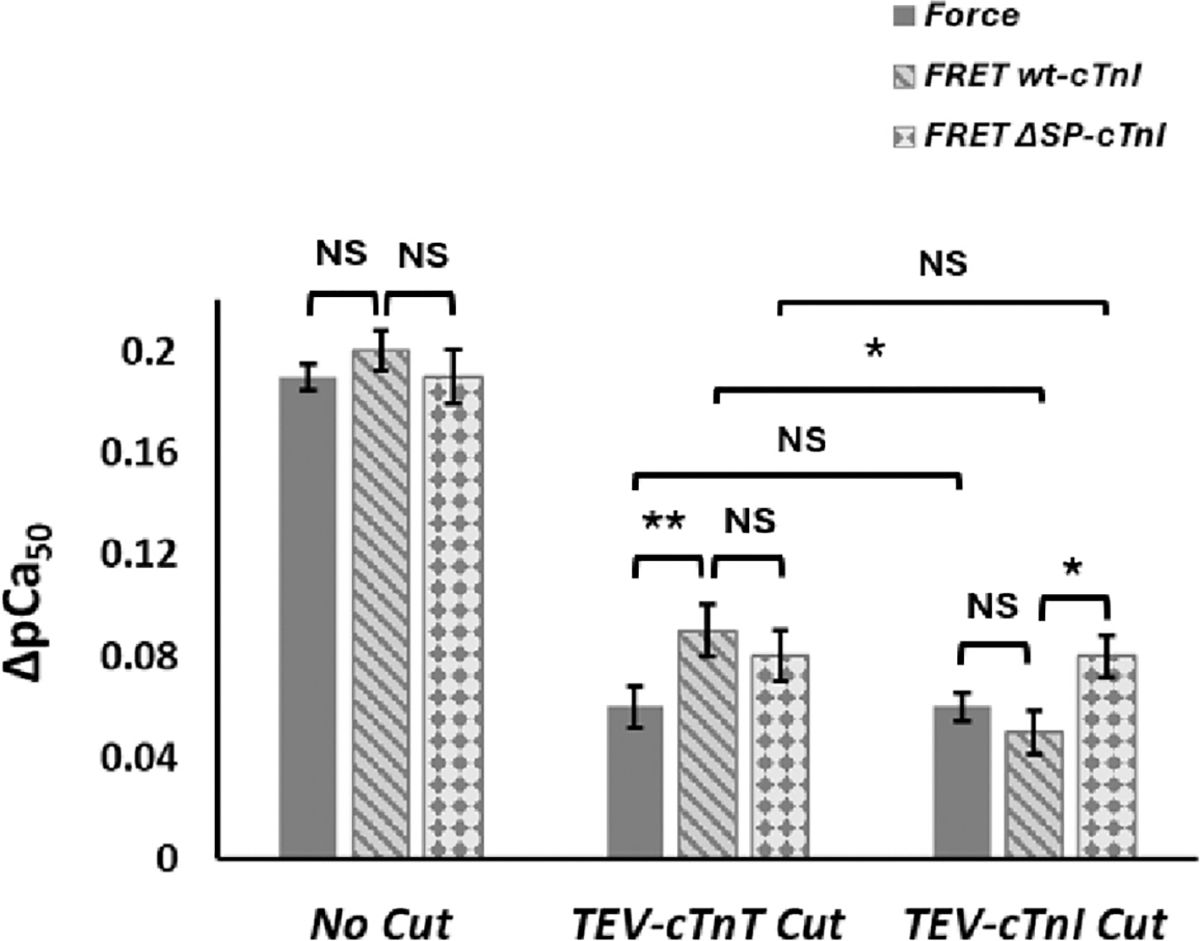
The pCa_50_ vs. force and FRET reconstituted with wt-cTnI (cMyc-tagged) and ΔSP-cTnI with no TEV cut, with TEV-cTnT cut, and with TEV-cTnI cut, respectively in short (filled bar) and long (diagonal stripes bar) SL (A). ΔpCa_50_ vs. force (filled bar) and Ca^2+^ sensitivity reconstituted with wt-cTnI (cMyc- tagged) (diagonal stripes bar) and ΔSP-cTnI (diamond grids bar) with no TEV cut, with TEV-cTnT cut, and with TEV-cTnI cut. Values are reported as mean ± SD. **, *p* < 0.01, *, *P* < 0.05, NS, no significant changes (B).

To compare the effects of TEV enzyme digestion of both TEV-cTnT and TEV-cMyc-cTnI on SL-induced ΔpCa_50_ changes, the results of changes in ΔpCa_50_ obtained by force- and FRET-Ca^2+^ titrations performed with different troponin reconstitution condition at different sarcomere lengths with/without TEV incubations are collectively shown in Fig. 6. The results show that TEV enzyme cutting both TEV-cTnT and TEV-cTnI cause a similar magnitude reduction in the SL-induced ΔpCa_50_ changes regardless of the presence of active myosin-actin XB interactions. In our design, the TEV-cTnI cut will separate the N-terminal region (1–80) of cTnI from the IT arm (Graph 1). Since the N-terminal region of cTnI is known to interact with the N-cTnC, it could play a role in transmitting the SL-stretch to the N-cTnC. The TEV-cTnI cut will break the link, thus blunting the SL effect.

## 3. Discussion

We have developed novel approaches to test the hypothesis that SL- induced increase in Ca^2+^ sensitivity of troponin/thin filament is attributed to the strong myosin-actin XB feedback via the cooperative activation mechanism [9,20]. Our use of FRET donor fluorescence intensity changes, which combined with the expression of a mutant cTnI permitted determinations of LDA while monitoring troponin signaling with normal and reduced numbers of strong XBs. Furthermore, to understand pathways in which SL-stretch enhances Ca^2+^ sensitivity of N- cTnC within the myocardial fibers we implemented TEV enzyme digestible TEV-cTnI and TEV-cTnT mutants to our reconstituted skinned myocardial fiber experiments. Several important findings were observed.

- Experiments using ΔSP-cTnI to block myosin-actin XB interaction led us to conclude that the increase in Ca^2+^ sensitivity of troponin in response to SL change from 1.8 μm to 2.2 μm is largely independent of the presence of force-generating actin-myosin interactions; thus, an alternative pathway exists to transmit stretch mechanics to increase Ca^2+^ sensitivity of myofilaments. Several alternative approaches are available in literature for blocking myosin-actin XB interaction, e.g., using cTnC mutant in which the Ca^2+^-binding site II is inactivated [63–66] or using blebbistatin as an inhibitor to prevent strong-actin-myosin interaction of skinned muscle fibers [35,75–77]. Compared to our ΔSP-cTnI approach, caveats with these alternative approaches are that either the cTnC or myosin head needs to be functionally deactivated by mutations or inhibitor binding; both can potentially undermine the proposed SL-induced myosin-troponin interaction of this study. In our experimental design, we used ΔSP-cTnI to separate troponin- Ca2+ binding from active actin-myosin interaction, and both troponin and myosin are functionally intact.
- Experiments using mavacamten to manipulate states myosin heads led us to conclude that even though active myosin-actin XB interactions are not essential for SL-induced increase in pCa_50_ of troponin, weakly bound (DRX) myosin heads are required for transmitting sarcomere strain to increase the Ca^2+^ sensitivity of the myofilament. This conclusion is based on the following arguments. The population of strong binding myosin heads is likely very small for the skinned muscle fibers reconstituted with troponin containing ΔSP-cTnI. Most of the myosin heads are expected in equilibrium of the population of DRX and the population of SRX. It is known that mavacamten stabilizes the SRX state by binding to myosin and reducing its availability for cross-bridge formation [78]. If the DRX myosin heads do participate in the proposed myosin-troponin interaction, applying mavacamten to the muscle fibers containing ΔSP-cTnI will shift the equilibrium of the populations of DRX and SRX to lower the level of DRX, thus negatively impact the observed SL-induced increase in pCa_50_ of troponin. In our study, 5 μM of mavacamten was used, which only suppressed muscle fiber force by 60 %. It suggests that under our experimental condition, there is still a significant population of DRX myosin heads that are available for the proposed potential interaction between troponin and myosin. Therefore, the observed blunting effect of mavacamten on the SL-induced increase in pCa_50_ of troponin is not maximal and it will depend on available level of DRX. There is evidence in support of this role of myosin in the DRX state in LDA. In studies with porcine ventricular strips, increased SL in the relaxed state was reported to induce an increase in the population of XBs in the DRX state [69]. This modification of the thick filament state, which was detected by loss of helical order of XB in X-ray diffraction experiments, has been interpreted to demonstrate an effect of titin stretch to strain the thick filament [69]. The increased population of DRX XBs- formation with stretch and length-dependent activation was significantly reduced by treatment with mavacamten and LDA was blunted. In a similar set of experiments that used different approaches involving a different sensor of troponin signaling and blebbistatin to inhibit strong XBs, Zhang et al. [35], also reported persistence of troponin-sensing by increased SL with and without strong XBs.
- Experiments using TEV enzyme digestible TEV-cTnI and TEV- cTnT mutants suggest that the Tm/actin-troponin interaction regions beyond the troponin core domain, specifically the interaction regions or beyond between Tm/actin and troponin structure containing residues 214–220 of cTnT, are likely involved in the SL-induced interaction with myosin heads, while the IT arm of the troponin and the N-terminus of cTnI are likely involved in transmitting the mechanic signal of SL stretching to the Ca^2+^ binding domain of cTnC. The T1 domain (residues 1–181) of cTnT, together with the N-terminal segment (residues 182 to 229) of T2 of cTnT, are known to anchor the core domain of troponin to actin filament by interacting with Tm on the surface of actin filament, thus, playing modulatory roles in myofilament regulation [79,80]. Our conclusion for this experiment is drawn from the following arguments. If the proposed troponin-myosin interaction is located at the core domain of the troponin or the IT arm region, TEV-cTnT cutting will have minimal effect on the SL- induced Ca^2+^ sensitivity changes in the presence of ΔSP-cTnI. If the proposed interaction occurs beyond the core domain, e.g., along the region of actin filament where cTnT-T1 and Tm bind, or the hinger region (residues 182 to 229) of cTnT-T2 anchoring the core domain of troponin to actin filament, TEV-cTnT cutting will either break the mechanic signal transmission to the core domain or interrupt the proposed interaction. Thus, either way will lead to a negative impact on the observed tSL-induced increase in pCa_50_ of troponin. The potential interactions between myosin and different regions of cTnT and cTnI have been investigated previously [81,82]. However, our previous in vitro sedimentation experiments showed binding of myosin to cTnI fragments containing residues 1–128 and T2 domain of cTnT, but not T1 domain of cTnT [81]. Since these earlier studies were performed with in vitro solution samples, further in situ experiments with skinned muscle fibers are needed in the future to reconcile this discrepancy and validate our hypothesis.

Although these findings shed new light on the mechanism of LDA, important questions remain. How in the absence of strong XBs is there transmission of a signal to the thin filament? Is the primary signal to the sarcomere imposed by stretch of titin reduced inter-filament spacing or altered thick filament strain? There are two prominent possibilities among the mechanisms that may be responsible for transmitting this strong XB independent signal to the thin filaments. One possible mechanism is the concept of SL-induced “myosin-troponin” interactions, i.e., so-called “troponin-bridge”. A second possible mechanism discussed below is the concept of a stretch induced modulation of mechano- sensitive mechanisms at the level of the thick filaments that promote an increased number of strong XBs as sarcomere are stretched. The “troponin-bridge” concept was first proposed based on results from a real-time X-ray diffraction study of stretch-activated Lethocerus indirect flight muscles (IFM), in which a delayed stretch activation was observed [43]. Later, a stretch-induced increase in the intensity of meridional reflection at 14.5 nm arising from troponin during diastole was also observed in a study of twitching rat cardiac muscle using similar technology, which also suggests a communication between myosin and troponin [36]. The potential signaling pathway of the myosin-troponin interaction may be related to titin-based regulation of inter-filament lattice spacing. Titin-based passive tension in response to cardiac myocyte stretching is known to positively engage in LDA via regulation of inter-filament lattice spacing [83,84] to favor myosin-actin XB interactions [85]. Even though the ratio of actin/troponin in a thin filament regulatory unit is 7 to 1, considering that extension of cTnT-T1 covers about 4 actins on the actin filament surface [21] and the high number of myosin heads distribute along each regulatory unit, it is very likely that SL-induced decreases in interfilament lattice spacing [41] allows several local myosin heads to reach over to troponin for interactions [86–88]. Thus, our results could be interpreted as providing evidence supporting this potential myosin-troponin interaction.

A second possible mechanism in LDA involves both thin and thick filament activation with a central role in the modulation of mechano- sensitive mechanisms in the thick filament [36,89]. Studies by Ait- Mou et al. [36] investigated this possibility in experiments comparing rat cardiac sarcomeres containing either native titin or giant titin (HM- titin) with high relative compliance. Passive tension and LDA were blunted in the HM-titin sarcomeres compared to the controls. When SL was increased, X-ray diffraction of the preparations revealed differences in the reflections related to X-ray signatures of thick filaments and to troponin with a blunting of modifications in reflections in the HM-titin sarcomeres compared to WT controls. The treatment of permeabilized myocytes with a fluorescence probe on cTnC with 2,3-butanedione monoxime (BDM) demonstrated the persistence of no effect of SL on Ca^2+^-binding affinity in both HM-titin sarcomeres and controls despite a significant depression in active tension development. There was however a diminished cTnC Ca^2+^-binding affinity in the HM-titin sarcomeres. These data indicated that the signal to the thin filament that increased pCa_50_ with titin stretch modified the transduction of the thin filament Ca^2+^-binding rather than altering its affinity. Other studies have also monitored thick and thin filament modifications with increased SL [35,90]. In this mechanism the initial binding of Ca^2+^ to cTnC induces stress via a few sentinel XBs on the thick filament that is cooperatively transmitted along the thick filament inducing an increase in DRX myosin motors that enter force generation as dictated by the prevailing Ca^2+^. These motors cooperatively activate the thin filament response to Ca^2+^as discussed above. However, a signal to activate the troponin complex can occur even with inhibition of strong XB binding to the thin filament. As reported here increases in sarcomere length may engage this mechanism when strong cross-bridges are inactivated as with treatment with blebbistatin or BDM.

Further evidence for this mechanism has been obtained in experiments in which probes on regulatory light chains have been used to monitor the movement of cross-bridges toward the thin filament with increased phosphorylation [91]. The use of these probes reporting RLC orientation in skinned fiber preparations has provided evidence that LDA activation involves both a thick filament sensing mechanism together with a thin filament sensing mechanism. Experimental manipulations indicated that the orientation of the RLC probe reports whether the XBs are in an off state folded back on the thick filament surface or in a state moving away from the thick filament. Under relaxing conditions at Ca^2+^ levels below the threshold for Ca^2+^-binding to cTnC increases in SL did not affect RLC orientation. However, with sub-maximal levels of Ca^2+^ the RLC probes reported a movement to the thin filament. These results were interpreted as demonstrating that in addition to mechano-sensing of the thick filament state thin filament activation is an essential element in the inter-filament signaling pathway. Contrasting data have been reported by Ma et al. [69], who monitored the off-state of XBs employing synchrotron small-angle X-ray diffraction in skinned porcine ventricular fibers. With increased SL at pCa 8, there was an increase in the population of DRX XBs available for interaction with thin filaments. The authors did not discuss the possible role of MyBP-C in these findings. They discussed possible reasons for the difference in their finding compared to intact rat trabeculae where there was no radial shift of myosin heads toward the thin filament with increases in SL.

In summary, our study demonstrated that the SL-induced changes in Ca^2+^ sensitivity of myocardial regulation is independent of strong myosin-actin XB interactions. Based on our experimental results and collective discussion above, we propose a strong myosin-actin XB independent model to account for the SL-induced Ca^2+^ activation of myofilament within the D-zone of sarcomere (Graph I). The core of the proposed model is the SL-induced potential direct interactions between weakly bound (DRX) myosin heads and troponin to initiate the mechanical stretch activation of the myocardium. During the myocardium stretch by ventricular filling, titin-based passive tension increases. This will lead to two consequences: *1)* the mechanical stretch alters the orientation of myosin heads at diastole and shifts myosin from population of SRX to DRX [92,93], thus, increasing the population of weakly bound myosin heads poised for entering force-generating XB interactions in subsequent systole [85]; and *2)* the stretch may decrease the interfilament spacing [41], thus, making it possible for some of the weakly bound myosin heads to overreach to troponin leading to potential interactions with an actin/Tm/troponin structural cluster composed of the cTnT-T1 domain and residues 182–229 of cTnT-T2 (see Graph 1). In our experimental design, the C-domains of ΔSP-cTnI are strongly bound to actin filament under all conditions, which makes it unlikely to interact with N-cTnC. Therefore, the mechanical effect sensed by the actin/Tm/troponin structural cluster is subsequently transmitted via the α-helix H1 structure (residues 43–79) of cTnI interacting with the C-cTnC domain [74], and the rest region of the N-terminus of cTnI, which in turn affects the N-cTnC-Ca^2+^ binding through allosteric modulation [94] and the cTnI switch peptide in a phosphorylation-dependent manner [95]. It is worth noting that the cardiac-specific N-terminus of cTnI and its phosphorylation states play important modulatory roles in contractile properties of heart muscle by altering Ca^2+^ sensitivity and LDA [44,96,97]. The proposed region (composed of residues 182–271 of cTnT) for transmitting SL-induced myosin interaction is critical to link the core domain of troponin to actin filament through multiple interactions of the T1 portion of cTnT with the Tm/actin filament and may play an important role in the cooperative activation mechanism of the myofilaments [9,20]. Since cardiac muscle thin filament activation appears to be sub-maximal under normal conditions [33,37,51,98,99], this SL-induced elevation in Ca^2+^-triggered thin filament activation could provide an effective and robust mechanism to enhance maximal force development in systole through the interaction between actin filament and large available population of myosin heads in DRX state. Although this study did not provide molecular evidence of the direct interaction between myosin heads and the proposed actin/Tm/cTnT cluster region, our approach provides a platform to test the proposed mechanism of mechanic signaling transduction by examining how cardiomyopathy mutations found in the Tm/cTnT-T1 region affect myofilament regulations [100]. For example, both HCM and DCM mutations in thin filament proteins are known to depress LDA of myofilament regulation and affect muscle force-Ca^2+^ sensitivity by either increasing Ca^2+^ sensitivity (HCM) or decreasing Ca^2+^ sensitivity [9,20]. Therefore, the proposed mechanism can be tested by examining whether the effects of HCM or DCM found in Tm/cTnT-T1 region on SL-induced Ca^2+^ sensitivity changes can be interrupted by TEV cuts. Blunted effects by TEV cuts will suggest the proposed mechanism is involved; otherwise, transmission of the cardiomyopathy mutation effects may involve strong myosin force development, which can be tested by using ΔSP-cTnI to block the transmission. Besides the involvement of myosin and the troponin complex in the proposed model, other myofilament proteins may also participate in the proposed mechanism. This proposed mechanism leaves open the question of the proposed role of MyBP-C in LDA. The binding sites of MyBP-C on the thin filament indicate that there is a role. We have found that the N-terminal domain, including C0-C2 domains, of MyBP-C plays important roles in proposed mechanisms of LDA. The results will be discussed in the next manuscript.

## 4. Materials and methods

### 4.1. Animal handling protocols

All the handling of our experimental animals followed the Institutional Animal Care and Use Committee at Washington State University and the Office of Laboratory Animal Welfare, National Institute of Health, Bethesda, MD. The cardiac muscle fibers preparation followed the established guidelines and protocol of ASAF #7212 approved by the Washington State University Institution Animal Care and Use Committee. Wild-type was anesthetized by 3 % isoflurane (Vet Pharma) in 95 % O_2_ and 5 % CO_2_ inhalation at a flow rate of 2 L/min. Hearts were immediately excised and placed into ice-cold dissecting solution.

### 4.2. Fiber dissection and skinning

Cardiac filament fibers were collected from left ventricular papillary tissues of 4- to 6-month-old mix gender adult Sprague Dawley (SD) rats by the following protocol that was established previously in our lab [34,101]. The heart was quickly removed from the rat while it was anesthetized with 2 min/L 3 % isoflurane and was immediately washed in the ice-cold high-relaxation (HR) buffer with 0.1 % protease inhibitor (Cat: 539134, EMD Millipore Corp.) (HR buffer: 50 mM BES, 30.83 mM K-propionate, 10 mM NaN_3_, 20 mM EGTA, 6.29 mM MgCl_2_, and 6.09 mM Na_2_ATP) by gentle finger pumps to wash the blood out. The left ventricular papillary bundle tissue was dissected out from the heart after wash and then cut to 150–200 μm diameter and 500 μm long uniformed size fibers in the HR buffer on a 4 °C cooling plate. Fibers were skinned by using the skinning buffer (1 % Triton X-100 in HR buffer) (Cat: T- 8532, Sigma) overnight at 4 °C with constant shaking.

### 4.3. Simultaneous measurement of isometric force and FRET fluorescence intensity in detergent-skinned cardiac muscle fibers

To study the sarcomere length effects on the thin filament regulation, the force contraction and steady-state FRET fluorescence intensity were measured during a calcium titration for both short and long sarcomeres. All the buffer recipes used in this experiment were from the previous paper published from this lab [62]. Reconstituted cardiac muscle fibers were mounted in between two clips inside the buffer chamber which were pre-filled with ice-cold pCa 9 buffer (50 mM BES, 5 mM NaN_3_, 2 mM EGTA, 5 mM NTA, 0.024 mM CaCl_2_, 6.87 mM MgCl_2_, 5.83 mM Na_2_ATP, and 71.14 mM K-propionate, 0.1 % protease inhibitor cocktails) [102]. The position of the fibers in the chamber were adjusted by tweezers and microscope. One clip was connected to a 5 mN level force transducer (SI Heidelberg KG7A, WPI), the other clip was connected to a mechanical motor which can be adjusted manually. The fiber can be stretched to different sarcomere lengths by the mechanical motor and the sarcomere length can be detected by a He—Ne laser with a diffraction method. The entire system of the fiber measurement was controlled at 4 °C by a circulatory cooling system (Cell MicroControls, CH). Calcium titration was generated by mixing the pCa 4.3 buffer (50 mM BES, 5 mM NaN_3_, 2 mM EGTA, 5 mM NTA, 10.11 mM CaCl_2_, 6.61 mM MgCl2, 5.83 mM Na_2_ATP, and 51 mM K-propionate, 0.1 % protease inhibitor cocktails) and pCa 9 buffer with a pre-set flowrate that continuously passes through the fiber chamber. Force contraction of the filaments was measured by the sensitive force transducer during the calcium titration. Following previous established protocol in our lab [103], the FRET fluorescence intensity changes of cTnC(13C/51C)_AEDENS-DDPM_ vs Ca^2+^ titration were monitored at emission peat of 510 nm with a 340 nm excitation from a LED source. The titration curves of force tension or fluorescence intensity changes vs Ca^2+^ concentrations were analyzed using a modified Hill equation to derive pCa_50_ and the Hill coefficient (n):

$$f=f_{0}+\frac{a{\left(10^{pCa}\right)}^{n}}{{\left(10^{pCa50}\;+\;10^{pCa}\right)}^{n}}$$

Where f indicates the normalized force or fluorescence intensity changes with pCa titration, and $f_{0}$ is the minimal y value. A represents the normalized maximum f, and n is Hill coefficient [85]. The accuracy of free [Ca^2+^] in the pCa titration was tested and calibrated by a Ca^2+^ chelator quin-2 solution. Quin-2 was used in this protocol to generate the correlation curve of Ca^2+^ concentration versus the pump flowrate of our titration system by measuring the fluorescence intensity of titration solution. Quin-2 forms a stable fluorescent complex with Ca^2+^ but not Mg^2+^ and has an excitation wavelength at 339 nm and a peak emission wavelength at 490 nm. 0.5 M Quin-2 stock solution was made in 0.1 M Tris buffer, pH 7.4 and stored in the 4 °C fridge. First, different concentrations of [Ca^2+^] from 0.024 mM to 10.11 mM every 2 mM was added to quin-2 solution and the fluorescence intensity was measured. Second, a Ca^2+^ titration with quin-2 buffer was applied with a certain pump flowrate in the titration system. The free [Ca^2+^] in the pCa buffer during titration was calculated and calibrated based on the paper Expanding the range of free calcium regulation [104]. The modified equation of the pump flowrate was customized and put into the excel sheet provided by the paper, and the free [Ca^2+^] in the pCa buffer titrated by our pump system can be calculated. Statistical significance is reported at *p* less than 0.05.

### 4.4. In vitro and in situ TEV enzyme digestion

For in vitro enzyme digestion, purified TEV-cTnT protein was stored in the 6 M urea CM buffer in −20 °C and was dialyzed against the reconstitution buffer and protein exchange buffer before use. The final concentration of the TEV-cTnT protein was measured by the spectrometer after dialysis to be 10 μM. 10 μL of TEV enzyme (Cat: Z03030, GenScript) was added into the protein solution at room temperature and the mixture was incubated in the 4 °C fridge for 4 h. For in situ enzyme digestion, skinned fibers were reconstituted with the two different troponin complexes respectively: 1) TEV-cTnT, wt-cTnI, and cTnC(13C/51C) _AEDANS-DDPM_ and 2) TEV-cTnI, wt-cTnT, and cTnC(13C/51C) AEDANS-DDPM. TEV-cTnT or TEV-cTnI protein was dialyzed with wt-cTnI and cTnC(13C/51C) _AEDANS-DDPM_ or wt-cTnT and cTnC(13C/51C) AEDANS-DDPM was dialyzed against urea and KCl buffer with same protocol described in Section 4. Wild type fibers were incubated in the protein solution with 0 M urea and 0.15 M KCl at 4 °C overnight. The fibers were exchanged with the troponin complex with TEV-cTnT and incubated with 10 μL TEV enzyme at 4 °C for 4 h. The enzyme digestion efficiency was tested by SDS PAGE gel and Western Blot assay.

### 4.5. Statistical data analysis

The two sample *t*-test with assuming unequal variances in Excel was used to analyze the statistical significance level of the ΔpCa between each condition. The conditions that compared in the analysis include (a) wt-cTnI and ΔSP-cTnI, (b) wt-MyBP-C and ΔN-MyBP-C, (c) with and without MAVA drug, and (d) with and without TEV digestion. Comparisons were made to determine whether the differences of ΔpCa between each condition are by chance. Fitted values of ΔpCa are reported as mean ± SEM with statistical significance level set at *P < 0.05 or **P < 0.01.

## Supplementary Material

1

## Figures and Tables

**Table 1:** Organization of pCa_50_, Hill coefficients (n), and ΔpCa_50_ values vs. Force and FRET for each condition.

Fiber	cTnI	XBs	Mava	Output	pCa_50_ (1.8)	nH	pCa_50_ (2.2)	nH	ΔpCa_50_

WT	WT	Yes	No	Force	5.77 ± 0.01	4.81 ± 0.011	5.98 ± 0.01	6.59 ± 0.012	0.21
	WT	Yes	No	FRET	5.74 ± 0.03	3.28 ± 0.014	5.95 ± 0.04	3.61 ± 0.025	0.21
			5 μM		5.61 ± 0.06	1.68 ± 0.026	5.72 ± 0.03	2.61 ± 0.016	0.11
	ΔSP	No	No	FRET	5.83 ± 0.05	1.75 ± 0.034	6.02 ± 0.06	1.82 ± 0.033	0.19
			5 μM		5.64 ± 0.05	2.26 ± 0.032	5.73 ± 0.04	2.47 ± 0.054	0.09

TEV		cTnI	XBs	Output	pCa_50_ (1.8)	nH	pCa_50_ (2.2)	nH	ΔpCa_50_

No digestion		WT	Yes	Force	5.72 ± 0.01	3.3 ± 0.023	5.91 ± 0.02	4.78 ± 0.041	0.19
				FRET	5.65 ± 0.05	2.3 ± 0.031	5.85 ± 0.04	3.14 ± 0.039	0.20
TEV		cTnI	XBs	Output	pCa_50_ (1.8)	nH	pCa_50_ (2.2)	nH	ΔpCa_50_
TEV-cTnT digestion		WT	Yes	Force	5.75 ± 0.01	2.32 ± 0.012	5.81 ± 0.01	3.15 ± 0.011	0.06
				FRET	5.73 ± 0.03	2.06 ± 0.025	5.8 ± 0.03	2.09 ± 0.032	0.07
		ΔSP	No	FRET	5.64 ± 0.04	2.25 ± 0.025	5.72 ± 0.05	1.87 ± 0.028	0.08

TEV		cTnI	XBs	Output	pCa_50_ (1.8)	nH	pCa_50_ (2.2)	nH	ΔpCa_50_

TEV-cTnI digestion		WT	Yes	Force	5.84 ± 0.02	4.1 ± 0.024	5.90 ± 0.02	3.61 ± 0.014	0.06
				FRET	5.84 ± 0.04	2.51 ± 0.059	5.89 ± 0.03	1.72 ± 0.031	0.05
		ΔSP	No	FRET	5.67 ± 0.05	1.61 ± 0.034	5.75 ± 0.04	1.63 ± 0.046	0.08

## Appendix A. Supplementary data

Supplementary data to this article can be found online at https://doi.org/10.1016/j.yjmcc.2025.03.003.
