# Supplementary material for: Myosin-actin crossbridge independent sarcomere length induced Ca2+ sensitivity changes in skinned myocardial fibers: Role of myosin heads
Source: J Mol Cell Cardiol. Author manuscript; Available in PMC 2026 Jun 1. (PMC13224722; doi:10.1016/j.yjmcc.2025.03.003)
Supplement: 1 [file NIHMS2172277-supplement-1.docx]

Supplementary Information

**Myosin-Actin Crossbridge Independent Sarcomere Length Induced Ca^2+^ Sensitivity Changes in Skinned Myocardial Fibers: Role of Myosin Heads**

Xutu Wang^1^, R. John Solaro^2^, Wen-Ji Dong^1,3^*

^1^Voiland School of Chemical and Bioengineering, Washington State University, Pullman, WA, 99163-1062, USA

^2^Department of Physiology and Biophysics, College of Medicine, University of Illinois at Chicago, Chicago, IL 60612-7342, USA.

^3^Department of Integrative Physiology and Neuroscience, Washington State University, Pullman, WA, 99163-1062, USA

**Materials and Methods**

1. *Protein and Peptide Preparations*

The recombinant cMyc-cTnI, ΔSP-cTnI, and wt-cTnT was each subcloned into a pET-3d vector and was expressed with BL21 competent cells (Cat: C600003, Invitrogen) and purified with CM and DEAE column following the protocol previously established in our lab [105].

The recombinant mutant cTnC(T13C/N51C) was labeled with double-cysteine from rat cDNA clones and was subcloned into a pET-3d vector, which was then used to transform BL21(DE3) cells (Invitrogen) and expressed under isopropyl β-D-1-thiogalactopyranoside (IPTG) (Sigma) induction. The expressed protein was purified as described before [34, 103, 106, 107]. The FRET donor 5-((((2-iodoacetyle)amino)ethyl)amino)naphthalene-1-sulfonic acid (IAEDANS) was added to purified cTnC(T13C/N51C) protein followed by our previously established protocol [105]. To separate the singly labeled cTnC(T13C/N51C) from unlabeled and doubly labeled proteins, the protein mixture went through the diethylaminoethyl column. The singly labeled cTnC(T13C/N51C)_AEDANSE_ was carefully collected and was labeled with the FRET donor N-(4-dimethylamino-3,5-dinitrophenyl) maleimide (DDPM) at the other cysteine to produce the the donor–acceptor labeled cTnC(T13C/N51C) AEDANS-DDPM. The labeling ratio of the donor-only sample was determined spectroscopically using ε_325_ = 6,000 cm−1 M−1 for AEDANS [34].

Each of 5 µL recombinant mutant TEV-cTnT, TEV-cMyc-cTnI, and TEV-SP-cTnI plasmid (GenScript, Piscataway, NJ) was transformed into 50 µL BL21(DE3) (Cat: C600003, Invitrogen,) on ice with a gentle mix and incubated on ice for 30 min. For each protein, the mixture was heat shocked in the 42 °C water bath for 20 sec and then was incubated on ice for another 5 min. 950 µL of room temperature S.O.C. medium (Cat: C600003, Invitrogen) was added into the mixture gently, and the mixture was incubated for 1 hour at 37 °C with 225 rpm shaking. 100 µL of the mixture was plated on each agar plate and spread well. After culturing the agar plates at 37 °C overnight, three colonies were picked from different locations and mixed in 20 mL LB medium (tryptone, yeast, NaCl) with 50 µM Carbenicillin (Cat: C46000, Research Products International) resistant for pre-culture. The 20 mL LB culture medium was cultured at 37 °C and 225 rpm shaking until the OD_600_ reading reached 1 (DU730, Beckman Coulter). The pre-culture was collected and spun down at 4000 xg for 15 min to get the cell pellet (Sorvall ST 16, ThermoScientific). The cell pellet was resuspended in fresh 400 mL LB medium in a 1:20 ratio. The bacteria were cultured until the OD_600_ reached 0.6-0.8, then 0.4 mM IPTG solution was added. After the IPTG induction, the culture medium was collected by centrifugation at 4000 xg again for 15 min after another 4 hours culture. The cell pellet was saved in -20 °C freezer for future lysis.

The frozen cell pellet was resuspended in a 6M urea lysis buffer (6 M urea, 30 mM Sodium citrate, 1mM EDTA, pH to 6 by citric acid) and was sonicated (Sonicator 3000, Misonix) on ice to lysis with 2 second active every 8 second interval for total one hour. Cell lysate was spun down for 30 min at 13,300 xg to remove the cell membrane and residue. The cleared supernatant was collected which contains the protein for future purification. The protein solution was dialyzed against a 6M urea CM buffer (6 M urea, 30 mM Sodium citrate, 1mM EDTA, pH to 6 by citric acid) overnight and loaded into the CM chromatography column (SP Sepharose Fast Flow Ion Exchange Chromatography Media, Cytiva, VWR) for purification. The CM column was equilibrated with ice-cold CM buffer (30 mM Sodium citrate, 1mM EDTA, pH to 6 by citric acid) for an hour before sample load. The column was washed with a CM buffer with 10 mM NaCl and then eluted with a 500 mM NaCl CM buffer gradient. Purified protein solution was collected and appropriately stored in -20 °C.

1. *Reconstitution of skinned cardiac fibers with fluorescently labeled cTnC(13C/51C) _AEDANS-DDPM_ wild-type and mutants of cTnI and cTnT.*

Skinned cardiac muscle fibers were incubated in the cTnC extraction buffer (5 mM CDTA, 0.6 mM NaN_3_, 40 mM Tris-HCl, 0.1% protease inhibitor cocktails, pH 8.4) at 4 °C for 2 to 3 hours with constant shaking (Rieck et al., 2013; Li et al., 2014). The fibers were washed twice in HR buffer at 4 °C for 5 min each after cTnC extraction and then were reconstituted in the protein exchange buffer (50 mM BES, 30.83 mM K-propionate, 5 mM NaN_3_, 10 mM EGTA, 6.09 mM Na-ATP, 10 mM BDM, 4 mM Ben-HCl, 1 mM DTT, 0.2 mM PMSF, 0.1% protease inhibitor cocktails, pH to 7 by KOH) with recombinant troponin protein complex at 4 °C. The concentration ratio for cTnI, wt-cTnT, and cTnC proteins in the reconstitution buffer was 1.2:1.2:1. Wt-cTnT and recombinant cTnC(13C/51C) _AEDENS-DDPM_ were reconstituted with cMyc-cTnI and ΔSP-cTnI each for different experiments. Recombinant troponin protein complex was dialyzed first against a gradient urea buffer from 6 M to 0 M, and it was dialyzed against a gradient KCl protein exchange buffer from 0.6 M to 0.2 M. Skinned fibers were incubated in the protein solution with 0M urea and 0.15 M KCl at 4 °C for overnight. Fibers were incubated in the protein exchange buffer for 4 hours twice to ensure the protein exchange efficiency reached 80%.

1. *Western blot assay*

The reconstitution efficiency was examined using Western blot analysis. Reconstituted fibers were washed 3 times in HR buffer at 4 °C and then incubated in the T-PER Tissue Protein Extraction Reagent (Cat: 78510, ThermoFisher) with 0.1% protease inhibitor cocktails for 15 min. Tissue was disturbed on ice with a tissue homogenizer (Model: 985370-395, BIOSPEC PRODUCTS, INC.) with on/5s and off/30s for 3 to 4 cycles until the tissue was fully homogenized. The protein extraction was added to the SDS 2x dye (Cat: LC1676, Novex) at 1:1 ratio then heat-shocked for 5 min at 95°C. The cMyc-cTnI and wt-cTnI protein bands were tested with anti-cTnI primary antibody (Cat: ab10231, Abcam, Waltham, MA) and HRP-labeled sheep anti-mouse secondary antibody (GE healthcare UK limited). cMyc-tagged wt-cTnI protein is 1 kDa heavier to the untagged wt-cTnI that can be separated by a 10% SDS-PAGE gel.

1. *Application of Mavacamten drug*

After skinning and protein exchanges, the fibers were incubated in the pCa 9 buffer with 5 µM mavacamten drug for 10 min before being mounted on the force measurement system. 5 µM mavacamten drug was added to both pCa 9 and pCa 4.3 buffers when the calcium titration was applied.

**Results**

1. *Western blot assay to examine efficiency of skinned myocardial fiber reconstitution with troponin containing TEV-cTnT and TEV-cTnI and TEV enzyme digestion*.


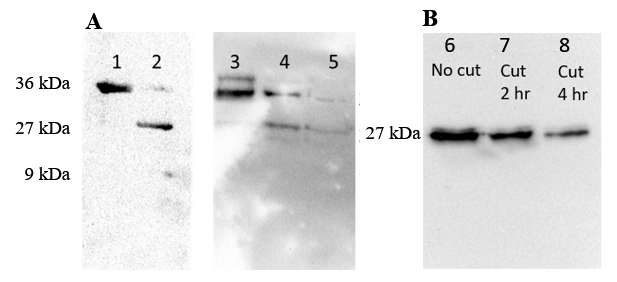
Before performing Ca^2+^ sensitivity experiments on the reconstituted fibers, we examined efficiency of *in situ* TEV digestions of TEV-cTnT and TEV-cTnI reconstituted myocardial fibers in HR buffer. Western blot analysis shown in Figure S1 suggests 74 ± 3.2 % for TEV-cTnT digestion and 79 ± 4% for TEV-cTnI digestion after 4-hr enzyme incubation of the fibers in the HR buffer. Western blot analysis was also performed on the HR buffer collected after enzyme digestion for detection of the T2- cTnT fragment band at 27 kDa. However, no discernible band was observed from lane 5, suggesting that cTnT and cTnI are all retained within the sarcomere structure of the skinned fibers after TEV digestion.

**Fig. S1.** Western blot analysis of *in vitro* and *in situ* TEV digestions of troponin complexes containing TEV-cTnT *(A)* and TEV-cMyc-cTnI *(B)*. Lane 2 shows the band of *In vitro* digestion of TEV in the troponin complex containing TEV-cTnT, cMyC-cTnI, and cTnC(13C/51C)_AEDANS-DDPM_. Compared to lane 1 (TEV-cTnT without digestion), a 4-hr TEV digestion (lane 2) of the sample led to disappearance of cTnT band at 36 kDa and a new cutting product band at ~27 kDa (matches to MW of the fragment of T2 - cTnT after cut), suggesting an effective in vitro TEV digestion of TEV-cTnT. Lane 3 - 5 show the band of *in situ* TEV digestion of fibers reconstituted with TEV-cTnT, cMyC-cTnI, and cTnC(13C/51C)_AEDANS-DDPM_. Lane 3 shows the band of reconstituted skinned fiber sample without TEV digestion, in which TEV-cTnT band at 36 kDa is clearly shown. Note: the band above the TEV-cTnT band at 36kDa is an unknown protein extracted from the myocardial fibers captured by cTnT polyclonal antibody (Cat: MBS311130, My BioSource) used in this experiment. Lane 4 shows the band after 4-hr TEV digestion of the reconstituted myocardial fibers, in which a weak band of the TEV-cTnT at 36 kDa was observed accompanied by a new band at 27 kDa, suggesting effective TEV digestions. Density analysis of the band at 36 kDa using ImageLab suggests > 75 % overall cut. Note: the 27 kDa band of TEV-cTnT fragment after digestion is weak because the antibody used in this experiment is a polyclonal antibody, and binding affinity became weak after target digestion. The HR buffer of the fibers after TEV digestion was collected and was tested with the same antibody shows very weak bands at both 36 and 27 kDa which indicate there were no significant leaking of cTnT proteins from fibers during the digestion. Lane 6 – 8 show the *in-situ* TEV digestion of TEV-cTnI reconstituted into skinned myocardial fibers. Monoclonal antibody (Cat: PA1-119, ThermoFisher) against TEV sequence was used in detecting TEV-cTnI. Lane 6: reconstituted skinned fiber sample without TEV digestion, in which a clear TEV-cTnI band at 27 kDa is shown. Lanes 7 and 8: after 2-hr and 4-hr digestion of the TEV-cTnI reconstituted fiber samples, respectively, the TEV-cTnI band at 27 kDa was significantly weakened. Density analysis using ImageLab suggests a 79% of TEV cut after 4-hr digestion.

1. *Skinned rat myocardial fibers reconstituted with the troponin complex containing TEV-cTnT, cMyc-cTnI, and cTnC(13C/51C)_AEDANSE-DDPM_ with and without TEV enzyme incubations*.

Figures S2 and S3 show the normalized changes in force and FRET donor fluorescence intensity vs. the Ca^2+^ titration at short and long SL without and with TEV digestion, respectively. The pCa_50_ and the Hill cooperativity coefficient (*n*) derived for each condition are listed in *Table 1*. Without TEV cut, the pCa_50_ obtained from FRET-Ca^2+^ titration was 5.65 ± 0.05 at 1.8 µm SL and 5.85 ± 0.04 at 2.2 µm SL, which is a ΔpCa_50_ 0.20 (Figure S2B). Similarly, the SL-induced changes in tension development titration yielded 0.19 change of ΔpCa_50_ (Figure S2A). These results are consistent with the results shown in Figure 3A and Figure 2B, respectively, suggesting TEV sequence insertion of cTnT does not alter the function of myocardial fibers. After TEV enzyme digestion in the presence of myosin-actin XB interactions, the SL-induced change in ΔpCa_50_ obtained from tension development vs. Ca^2+^ titration was reduced to 0.05 (Figure S3A). The troponin conformational change, monitored by FRET fluorescence titration, also became significantly less sensitive to Ca^2+^ (Figure S3B). The pCa_50_ at 1.8µm SL and 2.2µm SL was 5.73 ± 0.03 and 5.8 ± 0.03, respectively, which amounted to a 0.09 reduction in ΔpCa_50_. When the same FRET-Ca2+ titration experiments were performed with the skinned myocardial fibers reconstituted with the troponin complex containing TEV-cTnT, ΔSP-cTnI, and cTnC(13C/51C)_AEDANSE-DDPM_, in which the myosin-actin XB interaction was inhibited (Figure S3C), TEV enzyme incubation also reduced the SL-induced ΔpCa_50_ to 0.08. These results showed that TEV enzyme cutting induced the same level of the reduction of the SL-induced ΔpCa_50_ changes regardless of the presence of myosin-actin XB interaction. The results strongly suggest that the hinge region of cTnT where the TEV sequence was inserted plays an important role in the signal transmission of sarcomere stretch to the N-cTnC and modulation of the Ca^2+^ sensitivity of troponin regulation. All changes of pCa_50_ and the Hill coefficient (n) derived from fitting the titration curves at different SLs are given in Table 1.


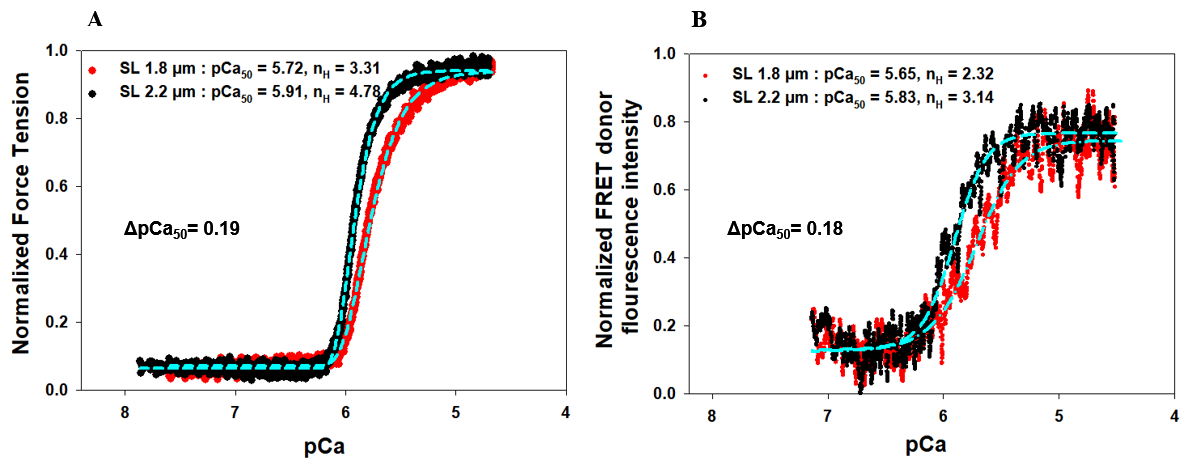


*
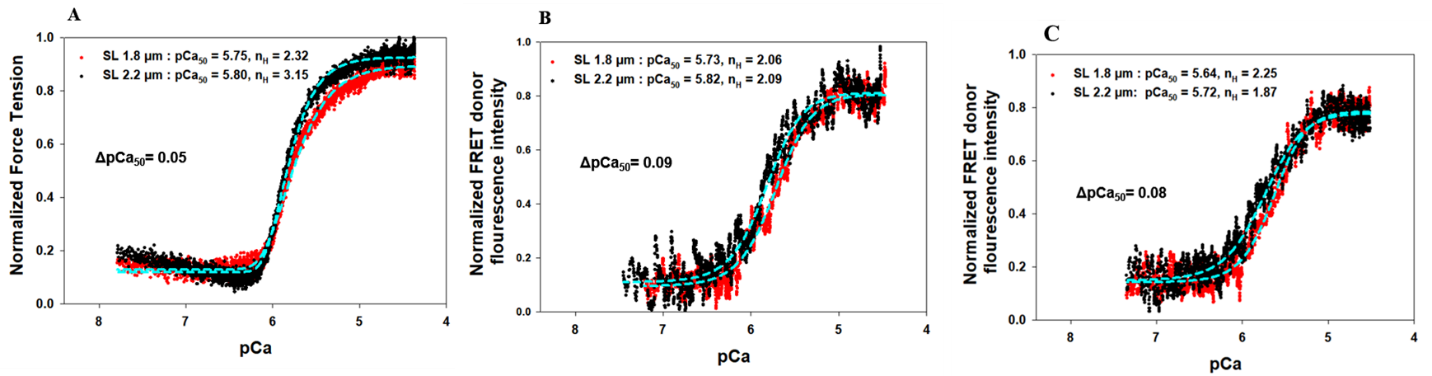
***Fig. S2**. The normalized changes in force (**A**) and FRET donor fluorescence intensity (**B**) vs. the Ca^2+^ titration at SL 1.8 (red) and 2.2 µm (black) of myocardial fibers reconstituted with troponin complex containing cTnC(13C/51C)_AEDANS-DDPM_, TEV-cTnT, and cMyc-cTnI. Data (dots) were fit to a 4 parameter Hill equation (cyan dash line) to extract the Ca^2+^ sensitivity (pCa_50_) and slope n_H_ (Hill coefficient) of the force-pCa relationship values at different SLs, which are listed in the legend. (N_fiber_ = 5 for A, and N_fiber_ = 6 for B, the curves shown in A and B represent the average curves of all measurements, and the errors calculated from STD are shown in Figure 6 and Table 3.)

**Fig. S3**. The normalized changes in force (**A**) and FRET donor fluorescence intensity vs. the Ca^2+^ titration at SL 1.8 (red) and 2.2 µm (black) of myocardial fibers reconstituted with troponin containing cTnC(13C/51C)_AEDANS-DDPM_, TEV-cTnT, and cMyc-cTnI (**B**) and reconstituted with troponin containing cTnC(13C/51C)_AEDANS-DDPM_, TEV-cTnT, and ΔSP-cTnI (**C**) in the presence of TEV cutting enzyme. Data (dots) in the figures were fit to a 4-parameter Hill equation (cyan dash line) to extract the Ca^2+^ sensitivity (pCa_50_) and slope n_H_ (Hill coefficient) of the force-pCa relationship values at different SLs, which are listed in the legend. (N_fiber_= 5 for A, N_fiber_=6 for B, and N_fiber_=7 for C, the curves shown in A, B, and C represent the average curves of all measurements, and the errors calculated from STD are shown in Figure 6 and Table 3.)

1. *Skinned myocardial fibers reconstituted with the troponin complex containing wt-cTnT, cTnC(13C/51C)_AEDANSE-DDPM_ and TEV-cMyc-cTnI or TEV-ΔSP-cTnI with TEV enzyme incubations*.

Figure S4 shows the changes in normalized tension development (A) and FRET donor fluorescence intensity (B) vs. Ca^2+^ titration at different SLs when TEV-cMyc-cTnI, which allows active myosin-actin XB interaction, was used for skinned myocardial fiber reconstitution. Compared to the untreated samples, the TEV enzyme incubation led to a reduction of SL-induced ΔpCa_50_ changes from 0.19~0.20 to 0.05~0.06 (comparing Figure S4A and Figure S4B to Figure 3), revealed by both force and FRET-Ca^2+^ titrations. When the XBs were inhibited in the reconstituted fibers containing TEV-ΔSP-cTnI, FRET-Ca^2+^ titration (Figure S4C) showed that the pCa_50_ of the TEV treated reconstituted fibers shifted from 5.84 to 5.67 at SL 1.8 and shifted from 6.02 to 5.75 at SL 2.2, respectively, suggesting a significant desensitization of Ca^2+^-N-cTnC binding at both short and long SL. SL induced ΔpCa_50_ also reduced from 0.19 to 0.08 (comparing Figure 3A to Figure S4C). The pCa_50_ and the Hill cooperativity coefficient (*n*) derived for each condition are listed in *Table 1*.


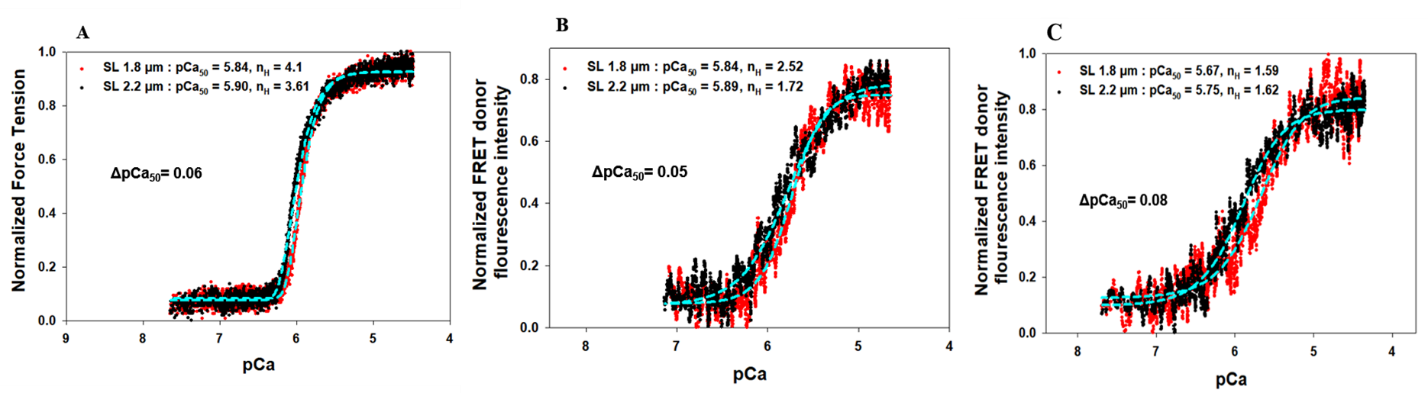


**Fig. S4**. The normalized changes in force tension (**A**) and FRET donor fluorescence intensity vs. the Ca^2+^ titration at SL 1.8 (red) and 2.2 µm (black) of myocardial fibers reconstituted with the troponin complex containing wt-cTnT, TEV-cMyc-cTnI, and cTnC(13C/51C)_AEDANS-DDPM_ (**B**) and reconstituted with the troponin complex containing wt-cTnT, TEV-ΔSP-cTnI,, and cTnC(13C/51C)_AEDANS-DDPM_ (**C**) in the presence of TEV enzyme. Data (dots) in the figures were fit to a 4 parameter Hill equation (dashed, cyan line) to extract the Ca^2+^ sensitivity (pCa_50_) and slope n_H_ (Hill coefficient) of the force-pCa relationship values in 1.8 and 2.2 µm SL, which are listed in the legend. (N_fiber_=7 for A, N_fiber_=8 for B, and N_fiber_=8 for C, the curves shown in A, B, and C represent the average curves of all measurements, and the errors calculated from STD are shown in Figure 6 and Table 3.)
